# Genomic prediction of relapse in recipients of allogeneic haematopoietic stem cell transplantation

**DOI:** 10.1038/s41375-018-0229-3

**Published:** 2018-08-08

**Authors:** J. Ritari, K. Hyvärinen, S. Koskela, M. Itälä-Remes, R. Niittyvuopio, A. Nihtinen, U. Salmenniemi, M. Putkonen, L. Volin, T. Kwan, T. Pastinen, J. Partanen

**Affiliations:** 1 Finnish Red Cross Blood Service, Helsinki, Finland; 2 Turku University Hospital, Turku, Finland; 3 Helsinki University Hospital, Comprehensive Cancer Center, Stem Cell Transplantation Unit, Helsinki, Finland; 4 McGill University, Montreal, Canada; 5 Children’s Mercy Kansas City, Kansas City, MO USA

**Keywords:** Cancer genomics, Genetics research, Cancer genetics

## Abstract

Allogeneic haematopoietic stem cell transplantation currently represents the primary potentially curative treatment for cancers of the blood and bone marrow. While relapse occurs in approximately 30% of patients, few risk-modifying genetic variants have been identified. The present study evaluates the predictive potential of patient genetics on relapse risk in a genome-wide manner. We studied 151 graft recipients with HLA-matched sibling donors by sequencing the whole-exome, active immunoregulatory regions, and the full MHC region. To assess the predictive capability and contributions of SNPs and INDELs, we employed machine learning and a feature selection approach in a cross-validation framework to discover the most informative variants while controlling against overfitting. Our results show that germline genetic polymorphisms in patients entail a significant contribution to relapse risk, as judged by the predictive performance of the model (AUC = 0.72 [95% CI: 0.63–0.81]). Furthermore, the top contributing variants were predictive in two independent replication cohorts (*n* = 258 and *n* = 125) from the same population. The results can help elucidate relapse mechanisms and suggest novel therapeutic targets. A computational genomic model could provide a step toward individualized prognostic risk assessment, particularly when accompanied by other data modalities.

## Introduction

Survival after allogeneic haematopoietic stem cell transplantation (allo-HSCT) as a treatment for malignancies of the blood and haematopoietic system is severely limited by relapse to the primary disease which occurs in approximately 30% of the patients depending on indication and stage of disease [1, 2]. The anti-neoplastic activity of grafted donor lymphocytes in the graft-versus-leukemia (GvL) effect is restrained by tumor immune evasion and immunosuppressive prophylactic medication necessitated by the lethal graft-versus-host disease (GvHD) [3, 4]. While the alloimmunity capacity of the graft is mainly governed by genetic matching of the human leukocyte antigen (HLA) loci [5], other germline genetic factors are also shown to contibute to rejection and GvL, most notably minor histocompatibility antigens [6, 7], donor-recipient mismatches in frequent gene deletions [8], as well as donor polymorphisms outside the HLA in genes regulating, e.g., immune response [9, 10]. Furthermore, particularly in the case of acute myeloid leukemia (AML), relapse risk is alleviated by donor haplotypes harboring higher numbers of activating killer-cell immunoglobulin-like receptors [11–13]. However, apart from the fundamental alloimmunity mechanisms, the significance of patient genetics to relapse remains to be studied in detail [14].

Defining the genetic architecture of complex traits has been pioneered by genome-wide association studies (GWASs). The GWAS approach considers the statistical significance of allele frequencies one locus at a time, accepting only *p*-values surpassing the genome-level correction for multiple testing, i.e., approximately 5 × 10^–8^ [15]. While adequately powered GWASs have discovered several important variants associated with multifactorial disorders and other complex phenotypes [16], the approach is not designed for predictive analysis as such. However, given the genetic component underlying many diseases including cancer [17], genetic information has the potential to improve and inform clinical decision making. In this regard, predictive genomics has been suggested to be of higher clinical value than simple associated markers [18]. As a way of complementing the classical GWAS approach, models relying on feature selection and machine learning methods aiming to identify a subset of variants with optimal predictive value have been developed and employed [19–22]. In combination with resampling statistics, these techniques allow modeling the effects of multiple variants together, deriving a genetic risk score with empirical error estimate and mining for potential synergistic functional interactions between variants and other factors.

In the present study, we have addressed the contribution of common germline single-nucleotide polymorphisms (SNPs) and small insertions and deletions (INDELs) to patient relapse risk by carrying out genome-wide sequencing of active immunoregulatory regions, the whole-exome and the full MHC region on 151 allo-HSCT recipients with HLA-matched sibling donors. To identify genetic variants affecting relapse susceptibility, we employ a machine learning approach by performing feature selection, Random forest classification model fitting, and evaluation of the predictive performance of the model through cross-validation. To further validate our approach, we test the predictive capability of the top variants in two independent cohorts of 258 and 125 sibling HSCT recipients from the same population.

## Patients and methods

### Acquisition of patient samples

The study cohort was originally composed of 161 HSCT patients with an HLA-matched sibling donor. Of the patients, 160 had relapse status information available, and 151 were diagnosed with a malignant disease. Relapse was defined as the recurrence of disease detected by clinical or molecular methods, thus both hematological and molecular relapses were taken into account. Detection of disease at any time point after HSCT was classified as relapse. The general characteristics of the study cohort are presented in Table 1. In summary, 48 recipients underwent allo-HSCT at Helsinki University Hospital during the years 2006–2011, and 113 recipients underwent allo-HSCT at Turku University Central Hospital during the years 2001–2015. The sibling pairs were matched with regard to the HLA-A, HLA-B, HLA-C, and HLA-DRB1 loci. The study was approved by the Ethics Committees of Helsinki University Central Hospital and Turku University Central Hospital, and the Finnish National Supervisory Authority for Welfare and Health. Additional details are provided in the Supplementary Methods.

**Table 1:** General characteristics of the discovery patient cohort

Clinical parameter		Value
Recipient age in years, median (range)		51 (3–70)
Donor age in years, median (range)		49 (7–72)
Donor-recipient gender, *n* (%)	Male-male	47 (29)
	Male-female	45 (28)
	Female-female	34 (21)
	Female-male	35 (22)
Diagnosis, *n* (%)	Acute myeloid leukemia	55 (34)
	Acute lymphoblastic leukemia	23 (14)
	Acute leukemia	3 (1)
	Chronic lymphocytic leukemia	8 (4)
	Chronic myelomonocytic leukemia	3 (1)
	Chronic myeloid leukemia	3 (1)
	Plasma cell leukemia	1 (1)
	T-cell prolymphocytic leukemia	1 (1)
	Non-Hodgkin’s lymphoma	9 (6)
	Hodgkin’s lymphoma	5 (3)
	Follicular lymphoma	1 (1)
	Mantle cell lymphoma	1 (1)
	Diffuse large B-cell lymphoma	1 (1)
	Multiple myeloma	12 (7)
	Myeloma	10 (6)
	Myelodysplastic syndrome	10 (6)
	Myelofibrosis	4 (2)
	Mastocytosis	1 (1)
	Chronic granulomatous disease	1 (1)
	Aplastic anemia^a^	9 (6)
Stem cell source, *n* (%)	Bone marrow	38 (24)
	Peripheral blood	121 (76)
Conditioning regimen, *n* (%)	Myeloablative	104 (65)
	Reduced intensity conditioning	57 (35)
CMV positive		113 (78)
aGvDH grades III–IV, *n* (%)		16 (10)
cGvHD, extensive, *n* (%)		52 (34)
Relapse, *n* (%)		49 (31)

*aGvHD* acute GVHD, *cGvHD* chronic GvHD, *CMV* cytomegalovirus, *GvHD* graft-versus-host disease

^a^Anemia diagnoses were omitted from analysis

### Genotyping

The discovery cohort was sequenced using a custom capture panel targeting the whole-exome, the full MHC region, and immune cell regulatory regions [23]. Quality filtering of the raw genotypes was performed by using the GATK best practices protocol [24] and thereafter comparing duplicated samples for overall genotype similarity at different DP and GQ parameter hard cutoff thresholds (Supplementary Fig. 1). The first Finnish independent replication cohorts was genotyped with Illumina Immunochip v1 (IC) and the Spanish cohort with Immunoarray v2.0 as described previously [25]. The second independent Finnish replication cohort was genotyped with Immunoarray v2.0 platform and was otherwise similarly processed as the first one. Additional details are available in the Supplementary Methods.

### Predictive model

**Fig. 1:**
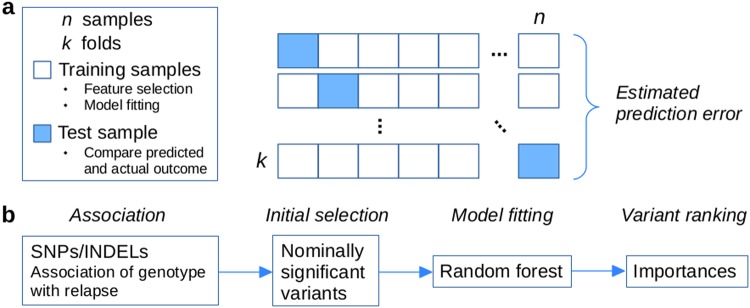
Schematic representation of the study setup. **a** Leave-one-out cross-validation (LOOCV) for feature selection and classification model fitting. Each sample is systematically left out in each fold. Prediction error estimates are based on left out samples (blue). **b** The analysis procedure within each LOOCV fold includes a first round of feature selection with a logistic regression association test followed by fitting a Random forest classification model on variants below an initial association *p*-value threshold

A first round of variant selection was performed with a logistic regression association test against relapse status using Plink v1.90b3u/v1.90b4.1 (www.cog-genomics.org/plink/1.9/) [26] with donor age, diagnosis, and graft type as covariates. Variants reaching a *p*-value < 0.001 were selected as inputs for the Random forest [27] classification model implemented in R software v3.3.3 library ranger v0.7.0 [28]. Both variant selection and Random forest model fitting were performed through leave-one-out cross-validation (LOOCV), and the prediction error estimate was calculated based on prediction of relapse status of samples left out from model fitting in each LOOCV fold (Fig. 1). The best predictive variants were selected using the importance metric of the Random forest model collected from the LOOCV folds and a permutation-based test. One-sided Mann–Whitney test and bootstrapped confidence intervals for the AUC were used for evaluating the predictive performance. Analysis of individual diagnoses and other additional details are available in the Supplementary Methods.

### Variant annotation

Colocalization of the top predictive variants with genes was examined using the ENSEMBL GRCh37 database. The list of genes associating with the top variants (Table 2) was queried against a number of public cancer gene databanks and annotated with the ToppGene (https://toppgene.cchmc.org/) [29] and PANTHER (http://pantherdb.org/tools) [30] annotation tools. Enrichment at FDR level < 0.05 was considered significant. Additional details are available in the Supplementary Methods.

**Table 2:** The top predictive variants and their associated genes

Chromosome	Position^a^	SNP ID	REF	ALT	ALT frequency	ENSEMBL gene ID	Gene symbol
1	228929158	rs4140409	C	T	0.675496689	NA	NA
1	228940615	rs241304	A	G	0.619205298	NA	NA
1	230244458	rs910500	A	G	0.440397351	ENSG00000143641	*GALNT2*
1	230245900	rs11585739	T	C	0.470198675	ENSG00000143641	*GALNT2*
1	230294715	rs4846913	C	A	0.506622517	ENSG00000143641	*GALNT2*
2	61070652	rs1432297	G	A	0.516556291	ENSG00000228414	*FLJ16341*
2	61072183	rs35194171	T	A	0.539735099	ENSG00000228414	*FLJ16341*
2	61072567	rs35741374	C	T	0.543046358	ENSG00000228414	*FLJ16341*
2	61075111	rs1177205	A	T	0.456953642	ENSG00000228414	*FLJ16341*
2	61075189	rs1177206	C	T	0.460264901	ENSG00000228414	*FLJ16341*
2	61075209	rs1177207	G	A	0.456953642	ENSG00000228414	*FLJ16341*
2	61075765	rs750026	T	C	0.463576159	ENSG00000228414	*FLJ16341*
2	61075987	rs750027	C	G	0.456953642	ENSG00000228414	*FLJ16341*
2	61080482	rs842625	G	A	0.456953642	ENSG00000228414	*FLJ16341*
2	61085723	rs842631	C	T	0.460264901	ENSG00000228414	*FLJ16341*
2	240674948	rs11678404	C	T	0.271523179	NA	NA
4	68311813	rs373609666	T	TACCGCCACCGCC	0.205298013	ENSG00000250075	*RP11–584P21.2*
6	3424481	rs9405201	C	T	0.32781457	ENSG00000137266	*SLC22A23*
6	3433318	rs17309827	T	G	0.400662252	ENSG00000137266	*SLC22A23*
6	3433713	rs9392492	G	GA	0.301324503	ENSG00000137266	*SLC22A23*
6	37789321	rs10456096	G	A	0.347682119	ENSG00000156639	*ZFAND3*
8	22865320	rs2430815	T	G	0.781456954	ENSG00000008853	*RHOBTB2*
8	81278885	rs12543811	G	A	0.586092715	NA	NA
10	64379326	rs2393904	C	T	0.387417219	ENSG00000138311	*ZNF365*
11	7720426	rs4367936	C	A	0.42384106	ENSG00000183378	*OVCH2*
11	30438948	rs492604	C	T	0.463576159	ENSG00000066382	*MPPED2*
13	77589725	rs599115	A	C	0.582781457	ENSG00000005812	*FBXL3*
16	56368689	rs1065375	C	T	0.5	ENSG00000087258	*GNAO1*
19	20735272	rs7251976	T	C	0.440397351	ENSG00000237440	*ZNF737*
20	61342535	rs35927656	T	C	0.374172185	ENSG00000101188	*NTSR1*
22	26168558	rs3848858	A	G	0.298013245	ENSG00000133454	*MYO18B*

^a^Chromosome position refers to GRCh37

### Replication

To evaluate the top SNPs with independent sets of patients, cohorts of 258 and 125 Finnish and 265 Spanish HSCT patients with a sibling donor genotyped with microarray platform were analyzed by fitting a Random forest model through LOOCV. None of these patients were included in the primary discovery cohort. The Spanish and the first Finnish cohorts have been described previously in detail [25]. The second Finnish cohort of 125 patients is described in the Supplementary Methods. The available SNPs in the first Finnish replication cohort in the order of numbers of missing genotypes are given in Supplementary Table 1. Additional details are available in the Supplementary Methods.

### Code availability

Code implenting the variant selection and model fitting via cross-validation is publicly available in GitHub (https://github.com/FRCBS/HSCT-relapse-model).

## Results

### Sequencing and variant calling

Samples from 161 recipients of haematopoietic stem cell transplantations were sequenced using a custom sequencing panel pipeline, encompassing the whole-exome, immune cell regulatory regions, and the full MHC segment. The pipeline yielded a median on-target coverage of 27.5× per sample. The GATK DepthOfCoverage tool applied to sample BAM files yielded a mean of 32.75 with standard deviation of 6.97 across all samples. The final quality filtering step was performed using a hard cutoff for the GQ parameter based on comparison of duplicates; the impact of varying GQ values on the similarity of duplicated samples is shown in Supplementary Fig. 1. At GQ > 18, the mean similarity was approximately 99%, resulting in an average of 32% of the candidate variants being discarded (Supplementary Fig. 1). Altogether, the quality filtered data contained 470,135 variants, of which 405,502 were SNPs, 68,721 were INDELs, and 2626 were others. After removing non-biallelic variants, a total of 437,679 variants was left.

### Covariate analysis

The genetic principal components were analyzed according to the variance explained by them; the eigenvalues reached a stable level at component five (Supplementary Fig. 2), and thus the first five components were included in the analysis. Correlation analysis between the covariates showed that batch and genetic principal components 1, 3, and 4 were intercorrelated with absolute Pearson’s coefficients ranging from 0.29 to 0.88 (Supplementary Fig. 3). Since the batches were from two different hospitals from different geographical locations, principal components 1, 3, and 4 likely reflected differing genetic backgrounds in the population. Donor and recipient ages had a correlation of 0.8. Furthermore, subject sex and donor-recipient sex direction of the transplant were associated, with absolute Pearson’s coefficients ranging from 0.49 to 0.65. After removing collinear variables (i.e., batch, recipient age, and transplant direction), the remaining variables were tested for association with relapse status. Out of these, diagnosis, graft type, donor age, and principal component 5 each had a nominally significant association (*p*-value < 0.1) with relapse status (Supplementary Table 1). Detailed analysis of PC5 revealed that its top loadings were solely from variants in the MHC region in chromosome 6, and thus were unlikely to indicate differences in the population structure. Finally, donor age, graft type, and diagnosis were included as covariates for association tests in the first round of variant selection with genetic association tests.

### Predictive performance

**Fig. 2:**
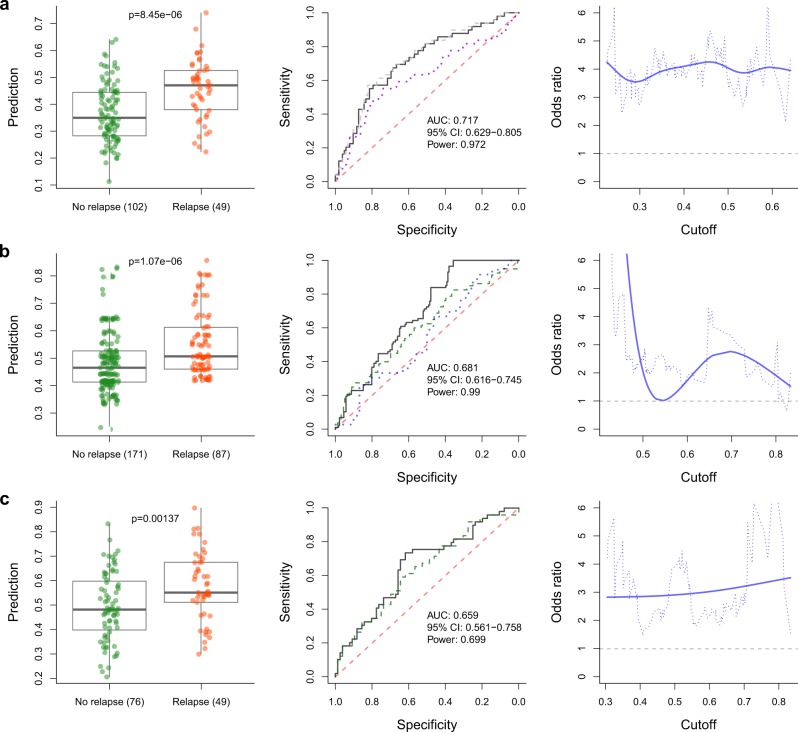
Estimated predictive performance of the model. The results from **a** the discovery dataset, and **b**–**c** the replication datasets. The left-hand side panels show the prediction value distributions over the LOOCV folds for the actual relapsed and non-relapsed groups by the Random forest classification model. The middle panels show the prediction ROC curves and AUC values. In **a**, the solid black ROC curve indicates the genetic model, the dashed gray curve indicates the model with principal components, and clinical and genetic variables, and the dotted purple curve shows the result using principal components and clinical data only. In **b**, the dashed green curve and the dotted blue curve show the results for allowing variants with <11 and <81 missing values, respectively. In **c**, the black curve and the dotted green curve show the results for higher (<0.3) and lower (<0.2) imputed genotype quality filtering stringencies, respectively. The right-hand side panels in **a**–**c** show the odds ratio for the correct prediction (*y*-axis) along the prediction model output values (*x*-axis). The *p*-values are calculated with one-sided Mann–Whitney test. The statistical power of the AUC is calculated at alpha level 0.01

The predictive performance of the model was estimated by comparing the distributions of LOOCV predictions between relapsed and non-relapsed groups, and by calculating the ROC/AUC values. The SNP/INDEL variant-based predictions with the Random forest model yielded a *p*-value of 8.45e-6 and an AUC of 0.717 (95% CI: 0.629–0.805) (Fig. 2a). The odds ratio of correct prediction was approximately 4 (Fig. 2a). When clinical covariates were included together with the genetic variants, a prediction performance *p*-value of 4.00e-6 and an AUC of 0.725 (95% CI: 0.638–0.8118) were obtained. When only the clinical covariates and PCs, without the genetic variants, were used for modeling, a prediction performance *p*-value of 0.0075 and an AUC of 0.623 (95% CI: 0.521–0.725) were obtained.

An independent cohort of 258 patients genotyped with the Immunochip platform [25] was used to evaluate the top predictors identified in the primary cohort. Altogether, 21 SNP/INDEL variants mapping to 8 different genes were found on the IC after genotype imputation (Supplementary Table 2). In 11 of these variants, the genotype was missing from at least one sample, ranging between 1 and 151 samples depending on the variant (Supplementary Table 2). Since a sample had to be removed if it had a missing genotype in any variant, inclusion of variants with missing values resulted in leaving increasingly more samples out. The numbers of variants and samples left after allowing for different numbers of missing values are given in Supplementary Table 3. The included variants were evaluated by fitting a Random forest classifier model via LOOCV. The prediction estimate yielded a *p*-value of 1.05e-06 and an AUC value of 0.681 (95% CI: 0.616–0.745) when variants with no missing values were included (Fig. 2b). When raising the threshold for the number of allowed missing values, the number of variants that could be included increased, but the prediction performance deteriorated in accordance with the number of missing values (Fig. 2b). Allowing for variants with less than 10 missing values yielded a prediction *p*-value of 0.004 and an AUC of 0.606 (95% CI: 0.528–0.683). Including variants with less than 50 missing values, the prediction *p*-value was 0.0036 and AUC 0.607 (95% CI: 0.530–0.684). Allowing for variants with less than 80 missing values yielded a prediction *p*-value of 0.226 and an AUC of 0.544 (95% CI: 0.432–0.657). We also tested replication in a cohort of 265 Spanish patients, but we did not obtain statistically significant results (data not shown).

A second Finnish cohort of 125 patients genotyped with the Immunoarray platform was analyzed to further evaluate the predictive capacity of the top variants in the Finnish population. To avoid the removal of samples due to missing data, probabilistic estimates of genotypes of imputed markers were used. The imputation quality filtering was implemented by applying standard deviation thresholds of <0.3 and <0.2, leaving 20 and 23 variants for analysis, respectively. The LOOCV modeling of data from the two quality thresholds yielded prediction *p*-values of 0.00137 and 0.00569, and AUC values of 0.659 (95% CI: 0.561–0.7575) and 0.6345 (95% CI: 0.5346–0.7345), respectively (Fig. 2c). Additional details are available in the Supplementary Material.

### Variant ranking and annotation

To evaluate which genes or genetic markers contributed most to the prediction, the variable importance metric values over the LOOCV folds were correlated against a permutation-based ranking metric from the whole dataset and plotted (Supplementary Figs. 4, 5). The correlation between the two ranking metrics was 0.91. The best predictors selected based on permutation and LOOCV importance are given in Table 2.

The genes colocalizing with the top predictive variants were functionally characterized by mining public databanks. Gene expression values in blood cancer cell lines and presence in cancer gene databases were determined (Supplementary Fig. 6a, Supplementary Table 4). Furthermore, a statistically significant representation of the genes in PubMed articles produced 50 significant results (Supplementary Fig. 6b, Supplementary Table 5). Finally, the genes with their significant (FDR < 0.05) interaction partners were tested for enrichment in Gene Ontology Biological Process functional categories. The results show that calcium signaling, epidermal growth factor, MAP kinase, and G-protein signaling were the pathways or functional groups with the highest fold enrichment values (Supplementary Fig. 6c, Supplementary Table 6).

### Analysis of individual diagnoses

Predictive analysis of AML patients as a separate group yielded a *p*-value of 0.000993 and an AUC of 0.767 (95% CI: 0.618–0.916) (Supplementary Fig. 7a). Replication of AML group in the Finnish cohort using eight top SNPs available on Immunochip yielded a *p*-value of 0.0721 and an AUC of 0.616 (95% CI: 0.469–0.764) (Supplementary Fig. 7b). Factorization of the full discovery cohort into diagnosis components showed that AUC varied between 0.613 and 1.00 depending on diagnosis (Supplementary Fig. 8). Additional details are available in the Supplementary Material.

## Discussion

The present study modeled the occurrence of relapse after allo-HSCT using genomic sequencing data in a predictive machine learning classification framework aiming to establish the level to which germline genetic variability in patients allows prediction of their relapse status. The principal finding of our analysis was that there is a statistically significant, albeit moderate, predictive relation between genetics and relapse occurrence, suggesting that common germline variability carries a risk for relapse in the allo-HSCT setting. Despite the relatively small sample size of our primary discovery cohort, the top SNP/INDEL variants also had predictive capacity in two independent sets of patients genotyped with microarray, testifying to their generalizability in the study population. However, the replication was limited to the polymorphisms shared between the two genotyping platforms. Inclusion of variants with missing genotype values reduced the predictive performance most likely owing to genotype imputation uncertainty. Moreover, failure to replicate the top variants in a different population could be due to differences in linkage disequilibrium structure, genetic background modifying variant effects, or treatment protocols.

The machine learning approach employed in this study is non-parametric and does not require the variables to be independent [27], making it suitable for modeling variants in linkage disequilibrium or otherwise correlated. Consistently with other studies on predictive genomics [21, 31], variants discovered through the machine learning approach do not necessarily surpass the univariate genome-wide level of significance of classical GWAS and could therefore help uncover hidden heritability [32] since the estimated genetic variance of many complex traits is mostly explained by a large number of common polymorphisms [33].

Treatment-related mortality can mask relapse occurrence, and consequently an underlying assumption in our analysis was that relapse is independent from death to, e.g., aGvHD or infection. Further, the diagnostically heterogeneous population in our study also implies that the results may be more representative of the most common diseases (i.e., AML and ALL) than others. However, the heterogeneity did not significantly manifest in the predictive performance as different diagnoses had relatively similar AUC values. This is consistent with our approach that aimed to identify variants independent of diagnosis in the discovery dataset.

In agreement with the used targeted sequencing approach, a majority of the top predictive variants mapped within genes, presenting potential candidates for studies on the molecular mechanisms of leukemia, drug development, relapse, and allo-HSCT. Together with their proteome interaction partners, the genes broadly represented ontologies involved in signaling of cell proliferation, differentiation, and apoptosis. Pathways such as MAPK and EGF together with G-protein and calcium secondary messenger signaling link various external stimuli to cellular growth and survival processes [34–37]. The remaining intergenic or non-coding RNA variants lacking specific annotation may still have regulatory roles in related processes [38]. However, as the current study was not to designed to address hypotheses on function, further research is required to clarify these questions. MHC region variants did not have significant predictive value, and HLA mismatching was not considered here due to extensive HLA matching between the sibling pairs.

Our results also showed that incorporating clinical and genetic PCA variables into the model improved predictive performance only marginally, and omitting the selected SNPs from the model led to markedly inferior predictive performance. This outcome likewise supports the relevance of genetic information for explaining the variation in susceptibility to relapse and is consistent with evidence of a genetic component underlying the risk for many common cancers [17]. To augment the genetic model, integrating different “omics” modalities such as somatic de novo mutations [39, 40], and transcriptomic [41], epigenetic [42, 43], and miRNA [44, 45] profiles could conceivably help achieve a predictive capability that adds substantial value to clinical decision making [40]. Furthermore, integrated modeling of the relationship between genetic variance, downstream molecular functions, and clinical endpoints is required to further understand how tumor phenotypes develop and acquire treatment-resistant properties.

In conclusion, the results presented here demonstrate the contribution of germline genetic variation to relapse occurrence in the allo-HSCT setting. However, further studies in different allo-HSCT populations, conditioning regimens, and other treatment factors are warranted. In the near future, the development of predictive models encompassing genomic and other molecular information hold the potential for improved clinical decision making and treatment optimization while helping reveal the molecular mechanisms underlying leukemic phenotypes.

## Electronic supplementary material

Supplementary Tables

Supplementary Methods

Supplementary Figures
