# Supplementary material for: Genomic prediction of relapse in recipients of allogeneic haematopoietic stem cell transplantation
Source: Leukemia. 2018 Aug 8;33(1):240–8. doi: 10.1038/s41375-018-0229-3 (PMC6326954; doi:10.1038/s41375-018-0229-3)
Supplement: Supplementary file 1 — Supplementary Tables [file 41375_2018_229_MOESM1_ESM.pdf]

Supplementary Table 1. Covariate associations with relapse status.

Variable	*Estimate of regression coefficient*	*Std. Error*	*z value*	*Pr(>|z|)*
(Intercept)	-63,4063	195,9441	-0,3236	0,7462
Txdate	0,0318	0,0975	0,3258	0,7446
DiagnosisAML	0,8973	0,7115	1,2613	0,2072
DiagnosisCLL	-14,6854	1167,8466	-0,0126	0,9900
DiagnosisMDS	-0,2831	1,0680	-0,2651	0,7909
DiagnosisMM	1,5377	1,0255	1,4995	0,1337
DiagnosisNHL	-0,4066	1,3120	-0,3099	0,7566
DiagnosisOther	1,3932	0,7998	1,7419	**0,0815**
Graft2	-1,1053	0,6260	-1,7657	**0,0774**
Conditioning2	0,6284	0,5937	1,0583	0,2899
sexM	-0,4667	0,4430	-1,0535	0,2921
CMVpos	0,4919	0,5794	0,8490	0,3959
dage	-0,0465	0,0178	-2,6070	**0,0091**
PCA1	3,1002	3,2041	0,9676	0,3333
PCA2	-3,8455	6,7746	-0,5676	0,5703
PCA3	6,0744	3,9043	1,5558	0,1197
PCA4	3,2989	3,4047	0,9689	0,3326
PCA5	-6,7920	3,2325	-2,1012	**0,0356**

Supplementary Table 2. SNPs on the Immunochip platform used for predictive analysis of the independent set of patients. The numbers of samples having missing genotypes are listed in the right-hand column.

SNP ID	***Number of samples with missing genotype***
rs11585739	0
rs1177205	0
rs1177206	0
rs1177207	0
rs1432297	0
rs2393904	0
rs35194171	0
rs35741374	0
rs750027	0
rs842631	0
rs17309827	1
rs910500	1
rs750026	2
rs842625	2
rs599115	4
rs4846913	8
rs9405201	8
rs492604	58
rs3848858	59
rs12543811	62
rs10456096	151

Supplementary Table 3. Impact of threshold of allowed number of missing genotypes on numbers of filtered variants and samples.

Threshold for allowed number of missing samples	Number of variants left	Number of samples left
0	11	258
10	17	236
50	17	236
80	20	112

Supplementary Table 4. ToppGene PubMed enrichment results for the genes associated with the top predictive variants. Different topics are grouped and highlighted with corresponding colours.

ID	*Name*	*Source*	*pValue*	*FDR B&H*	*FDR B&Y*	*Bonferroni*	*Genes from Input*	*Genes in Annotation*
21072187	Evidence for significant overlap between common risk variants for Crohn's disease and ankylosing spondylitis.	Pubmed	3.027E-5	9.398E-4	6.141E-3	1.168E-2	2	31
19760754	Analysis of 39 Crohn's disease risk loci in Swedish inflammatory bowel disease patients.	Pubmed	4.817E-5	9.398E-4	6.141E-3	1.859E-2	2	39
20930524	Ectopic expression of RhoBTB2 inhibits migration and invasion of human breast cancer cells.	Pubmed	2.654E-4	9.398E-4	6.141E-3	1.024E-1	1	1
26060304	Progressive Movement Disorder in Brothers Carrying a GNAO1 Mutation Responsive to Deep Brain Stimulation.	Pubmed	2.654E-4	9.398E-4	6.141E-3	1.024E-1	1	1
10690540	Neurotensin receptor-1 mRNA analysis in normal pancreas and pancreatic disease.	Pubmed	2.654E-4	9.398E-4	6.141E-3	1.024E-1	1	1
26334593	Epigenetic silencing of NTSR1 is associated with lateral and noninvasive growth of colorectal tumors.	Pubmed	2.654E-4	9.398E-4	6.141E-3	1.024E-1	1	1
20980811	RhoBTB2 (DBC2) comes of age as a multifunctional tumor suppressor.	Pubmed	2.654E-4	9.398E-4	6.141E-3	1.024E-1	1	1
14962098	Sezary syndrome cells unlike normal circulating T lymphocytes fail to migrate following engagement of NT1 receptor.	Pubmed	2.654E-4	9.398E-4	6.141E-3	1.024E-1	1	1
22152306	Heterozygosity for a loss-of-function mutation in GALNT2 improves plasma triglyceride clearance in man.	Pubmed	2.654E-4	9.398E-4	6.141E-3	1.024E-1	1	1
17653899	Mutation analysis of the DBC2 gene in sporadic and familial breast cancer.	Pubmed	2.654E-4	9.398E-4	6.141E-3	1.024E-1	1	1
26902265	Neurotensin-induced miR-133α expression regulates neurotensin receptor 1 recycling through its downstream target aftiphilin.	Pubmed	2.654E-4	9.398E-4	6.141E-3	1.024E-1	1	1
8078296	Association of heterotrimeric GTP binding regulatory protein (Go) with mitosis.	Pubmed	2.654E-4	9.398E-4	6.141E-3	1.024E-1	1	1
23807075	No association of neurotensin receptor 1 gene polymorphisms with coping styles in healthy Chinese-Han individuals.	Pubmed	2.654E-4	9.398E-4	6.141E-3	1.024E-1	1	1
21394204	Neurotensin receptor 1 gene (NTSR1) polymorphism is associated with working memory.	Pubmed	2.654E-4	9.398E-4	6.141E-3	1.024E-1	1	1
1556134	Mutagenesis of the amino terminus of the alpha subunit of the G protein Go. In vitro characterization of alpha o beta gamma interactions.	Pubmed	2.654E-4	9.398E-4	6.141E-3	1.024E-1	1	1
23912186	Initiation of GalNAc-type O-glycosylation in the endoplasmic reticulum promotes cancer cell invasiveness.	Pubmed	2.654E-4	9.398E-4	6.141E-3	1.024E-1	1	1
22551944	The role of cholesterol on the activity and stability of neurotensin receptor 1.	Pubmed	2.654E-4	9.398E-4	6.141E-3	1.024E-1	1	1
7705473	Up-regulation in late pregnancy of both Go1 alpha and Go2 alpha isoforms in human myometrium.	Pubmed	2.654E-4	9.398E-4	6.141E-3	1.024E-1	1	1
28747448	Movement disorder in GNAO1 encephalopathy associated with gain-of-function mutations.	Pubmed	2.654E-4	9.398E-4	6.141E-3	1.024E-1	1	1
22351618	Common variants at the 19p13.1 and ZNF365 loci are associated with ER subtypes of breast cancer and ovarian cancer risk in BRCA1 and BRCA2 mutation carriers.	Pubmed	2.654E-4	9.398E-4	6.141E-3	1.024E-1	1	1
23546941	Decreased expression of the DBC2 gene and its clinicopathological significance in breast cancer: correlation with aberrant DNA methylation.	Pubmed	2.654E-4	9.398E-4	6.141E-3	1.024E-1	1	1
21278746	Common variants in ZNF365 are associated with both mammographic density and breast cancer risk.	Pubmed	2.654E-4	9.398E-4	6.141E-3	1.024E-1	1	1
12911629	A novel function of Goalpha: mediation of extracellular signal-regulated kinase activation by opioid receptors in neural cells.	Pubmed	2.654E-4	9.398E-4	6.141E-3	1.024E-1	1	1
9442070	The glycosylation and structure of human serum IgA1, Fab, and Fc regions and the role of N-glycosylation on Fcα receptor interactions.	Pubmed	2.654E-4	9.398E-4	6.141E-3	1.024E-1	1	1
9852147	Recycling of golgi-resident glycosyltransferases through the ER reveals a novel pathway and provides an explanation for nocodazole-induced Golgi scattering.	Pubmed	2.654E-4	9.398E-4	6.141E-3	1.024E-1	1	1
26471393	Potent antitumor effect of neurotensin receptor-targeted oncolytic adenovirus co-expressing decorin and Wnt antagonist in an orthotopic pancreatic tumor model.	Pubmed	2.654E-4	9.398E-4	6.141E-3	1.024E-1	1	1
23993195	De Novo mutations in GNAO1, encoding a Gαo subunit of heterotrimeric G proteins, cause epileptic encephalopathy.	Pubmed	2.654E-4	9.398E-4	6.141E-3	1.024E-1	1	1
19937980	A novel tumor suppressor gene RhoBTB2 (DBC2): frequent loss of expression in sporadic breast cancer.	Pubmed	2.654E-4	9.398E-4	6.141E-3	1.024E-1	1	1
23110888	The NTSR1 gene modulates the association between hippocampal structure and working memory performance.	Pubmed	2.654E-4	9.398E-4	6.141E-3	1.024E-1	1	1
26287344	[Association between ZNF365 gene polymorphisms and bronchial asthma in children].	Pubmed	2.654E-4	9.398E-4	6.141E-3	1.024E-1	1	1
28025493	Analysis to Estimate Genetic Variations in the Idarubicin-Resistant Derivative MOLT-3.	Pubmed	2.654E-4	9.398E-4	6.141E-3	1.024E-1	1	1
25778778	Lack of replication for the myosin-18B association with mathematical ability in independent cohorts.	Pubmed	2.654E-4	9.398E-4	6.141E-3	1.024E-1	1	1
24740203	Single-nucleotide polymorphisms in SLC22A23 are associated with ulcerative colitis in a Canadian white cohort.	Pubmed	2.654E-4	9.398E-4	6.141E-3	1.024E-1	1	1
8995438	Characterization of the genomic structure, promoter region, and a tetranucleotide repeat polymorphism of the human neurotensin receptor gene.	Pubmed	2.654E-4	9.398E-4	6.141E-3	1.024E-1	1	1
22992780	Polypeptide N-acetylgalactosaminyltransferase 2 regulates cellular metastasis-associated behavior in gastric cancer.	Pubmed	2.654E-4	9.398E-4	6.141E-3	1.024E-1	1	1
24401289	Neurotensin receptor 1 gene polymorphisms are associated with personality traits in healthy Chinese individuals.	Pubmed	2.654E-4	9.398E-4	6.141E-3	1.024E-1	1	1
25159184	Association of Neurotensin receptor 1 gene polymorphisms with processing speed in healthy Chinese-Han subjects.	Pubmed	2.654E-4	9.398E-4	6.141E-3	1.024E-1	1	1
25177240	Identification of plasma biomarker candidates in glioblastoma using an antibody-array-based proteomic approach.	Pubmed	2.654E-4	9.398E-4	6.141E-3	1.024E-1	1	1
26239958	GALNT2 mRNA levels are associated with serum triglycerides in humans.	Pubmed	2.654E-4	9.398E-4	6.141E-3	1.024E-1	1	1
24582885	GALNT2 enhances migration and invasion of oral squamous cell carcinoma by regulating EGFR glycosylation and activity.	Pubmed	2.654E-4	9.398E-4	6.141E-3	1.024E-1	1	1
27071848	Glycomimetics Targeting Glycosyltransferases: Synthetic, Computational and Structural Studies of Less-Polar Conjugates.	Pubmed	2.654E-4	9.398E-4	6.141E-3	1.024E-1	1	1
23117232	Expression of GALNT2 in human extravillous trophoblasts and its suppressive role in trophoblast invasion.	Pubmed	2.654E-4	9.398E-4	6.141E-3	1.024E-1	1	1
17906984	Genetic analysis of the DBC2 gene in gastric cancer.	Pubmed	2.654E-4	9.398E-4	6.141E-3	1.024E-1	1	1
7527372	A WAGR region gene between PAX-6 and FSHB expressed in fetal brain.	Pubmed	2.654E-4	9.398E-4	6.141E-3	1.024E-1	1	1
18809332	Novel insights into GPCR-peptide interactions: mutations in extracellular loop 1, ligand backbone methylations and molecular modeling of neurotensin receptor 1.	Pubmed	2.654E-4	9.398E-4	6.141E-3	1.024E-1	1	1
24357116	Neurotensin receptor1 antagonist SR48692 reduces proliferation by inducing apoptosis and cell cycle arrest in melanoma cells.	Pubmed	2.654E-4	9.398E-4	6.141E-3	1.024E-1	1	1
9763490	Correlative ultrastructural distribution of neurotensin receptor proteins and binding sites in the rat substantia nigra.	Pubmed	2.654E-4	9.398E-4	6.141E-3	1.024E-1	1	1
25748484	A novel syndrome of Klippel-Feil anomaly, myopathy, and characteristic facies is linked to a null mutation in MYO18B.	Pubmed	2.654E-4	9.398E-4	6.141E-3	1.024E-1	1	1
15305387	Reduced expression of MYO18B, a candidate tumor-suppressor gene on chromosome arm 22q, in ovarian cancer.	Pubmed	2.654E-4	9.398E-4	6.141E-3	1.024E-1	1	1
23483448	Association between neurotensin receptor 1 (NTR1) gene polymorphisms and schizophrenia in a Han Chinese population.	Pubmed	2.654E-4	9.398E-4	6.141E-3	1.024E-1	1	1

19	Cancer/Chemotherapy
4	Autoimmune
2	Immunology
3	Other diseases

Supplementary Table 5. ToppGene interaction results for the genes associated with the top predictive variants.

ID	*Name*	*pValue*	*FDR B&H*	*FDR B&Y*	*Bonferroni*	*Genes from Input*	*Genes in Annotation*
int:FAM72A	FAM72A interactions	1.170E-3	4.009E-2	2.265E-1	1.861E-1	1	2
int:NTSR1	NTSR1 interactions	1.755E-3	4.009E-2	2.265E-1	2.790E-1	1	3
int:GRM6	GRM6 interactions	1.755E-3	4.009E-2	2.265E-1	2.790E-1	1	3
int:ZNF365	ZNF365 interactions	2.339E-3	4.009E-2	2.265E-1	3.720E-1	1	4
int:MYO18B	MYO18B interactions	2.339E-3	4.009E-2	2.265E-1	3.720E-1	1	4
int:ZFAND3	ZFAND3 interactions	2.339E-3	4.009E-2	2.265E-1	3.720E-1	1	4
int:PDE6H	PDE6H interactions	2.923E-3	4.009E-2	2.265E-1	4.648E-1	1	5
int:FFAR2	FFAR2 interactions	2.923E-3	4.009E-2	2.265E-1	4.648E-1	1	5
int:HRH4	HRH4 interactions	2.923E-3	4.009E-2	2.265E-1	4.648E-1	1	5
int:ADRA2C	ADRA2C interactions	3.507E-3	4.009E-2	2.265E-1	5.576E-1	1	6
int:PTPN1	PTPN1 interactions	3.688E-3	4.009E-2	2.265E-1	5.864E-1	2	159
int:CD69	CD69 interactions	4.091E-3	4.009E-2	2.265E-1	6.504E-1	1	7
int:CAGE1	CAGE1 interactions	4.674E-3	4.009E-2	2.265E-1	7.431E-1	1	8
int:SCN8A	SCN8A interactions	4.674E-3	4.009E-2	2.265E-1	7.431E-1	1	8
int:RGS5	RGS5 interactions	4.674E-3	4.009E-2	2.265E-1	7.431E-1	1	8
int:GPR35	GPR35 interactions	4.674E-3	4.009E-2	2.265E-1	7.431E-1	1	8
int:JSRP1	JSRP1 interactions	5.257E-3	4.009E-2	2.265E-1	8.358E-1	1	9
int:CRHR1	CRHR1 interactions	5.839E-3	4.009E-2	2.265E-1	9.284E-1	1	10
int:NTS	NTS interactions	5.839E-3	4.009E-2	2.265E-1	9.284E-1	1	10
int:RGS9	RGS9 interactions	5.839E-3	4.009E-2	2.265E-1	9.284E-1	1	10
int:S1PR5	S1PR5 interactions	5.839E-3	4.009E-2	2.265E-1	9.284E-1	1	10
int:RGS1	RGS1 interactions	6.421E-3	4.009E-2	2.265E-1	1.000E0	1	11
int:PDE6G	PDE6G interactions	6.421E-3	4.009E-2	2.265E-1	1.000E0	1	11
int:MPPED2	MPPED2 interactions	6.421E-3	4.009E-2	2.265E-1	1.000E0	1	11
int:PIANP	PIANP interactions	7.003E-3	4.009E-2	2.265E-1	1.000E0	1	12
int:ADORA1	ADORA1 interactions	7.003E-3	4.009E-2	2.265E-1	1.000E0	1	12
int:SULT1A3	SULT1A3 interactions	7.585E-3	4.009E-2	2.265E-1	1.000E0	1	13
int:RGS10	RGS10 interactions	7.585E-3	4.009E-2	2.265E-1	1.000E0	1	13
int:RFX2	RFX2 interactions	8.166E-3	4.009E-2	2.265E-1	1.000E0	1	14
int:ADRA2A	ADRA2A interactions	8.166E-3	4.009E-2	2.265E-1	1.000E0	1	14
int:FBXL3	FBXL3 interactions	8.166E-3	4.009E-2	2.265E-1	1.000E0	1	14
int:RGS4	RGS4 interactions	8.747E-3	4.009E-2	2.265E-1	1.000E0	1	15
int:SLC22A23	SLC22A23 interactions	8.747E-3	4.009E-2	2.265E-1	1.000E0	1	15
int:RGS7	RGS7 interactions	9.328E-3	4.009E-2	2.265E-1	1.000E0	1	16
int:GNG3	GNG3 interactions	9.328E-3	4.009E-2	2.265E-1	1.000E0	1	16
int:RGS16	RGS16 interactions	9.328E-3	4.009E-2	2.265E-1	1.000E0	1	16
int:GABBR1	GABBR1 interactions	9.328E-3	4.009E-2	2.265E-1	1.000E0	1	16
int:PCP2	PCP2 interactions	9.908E-3	4.040E-2	2.282E-1	1.000E0	1	17
int:GPSM2	GPSM2 interactions	9.908E-3	4.040E-2	2.282E-1	1.000E0	1	17
int:NGB	NGB interactions	1.049E-2	4.067E-2	2.298E-1	1.000E0	1	18
int:CACNA1B	CACNA1B interactions	1.049E-2	4.067E-2	2.298E-1	1.000E0	1	18
int:GALNT2	GALNT2 interactions	1.107E-2	4.072E-2	2.301E-1	1.000E0	1	19
int:RGS14	RGS14 interactions	1.165E-2	4.072E-2	2.301E-1	1.000E0	1	20
int:BTG2	BTG2 interactions	1.165E-2	4.072E-2	2.301E-1	1.000E0	1	20
int:PAXBP1	PAXBP1 interactions	1.165E-2	4.072E-2	2.301E-1	1.000E0	1	20
int:DSCC1	DSCC1 interactions	1.223E-2	4.072E-2	2.301E-1	1.000E0	1	21
int:RGS19	RGS19 interactions	1.281E-2	4.072E-2	2.301E-1	1.000E0	1	22
int:SLC30A4	SLC30A4 interactions	1.281E-2	4.072E-2	2.301E-1	1.000E0	1	22
int:RHOBTB2	RHOBTB2 interactions	1.281E-2	4.072E-2	2.301E-1	1.000E0	1	22
int:AP4S1	AP4S1 interactions	1.281E-2	4.072E-2	2.301E-1	1.000E0	1	22

Supplementary Table 6. Gene Ontology Biological Process enrichment analysis results from PANTHER for the genes and their significant interactions.

GO biological process complete	*Homo sapiens - REFLIST (21042)*	*Input (50)*	*Input (expected)*	*Fold Enrichment*	*P-value (Bonferroni)*
positive regulation of epidermal growth factor-activated receptor activity (GO:0045741)	12	3	.03	> 100	3.08E-02
positive regulation of epidermal growth factor receptor signaling pathway (GO:0045742)	37	5	.09	56.87	2.88E-04
positive regulation of ERBB signaling pathway (GO:1901186)	39	5	.09	53.95	3.74E-04
negative regulation of calcium ion transmembrane transport (GO:1903170)	38	4	.09	44.30	1.98E-02
positive regulation of protein tyrosine kinase activity (GO:0061098)	44	4	.10	38.26	3.53E-02
regulation of G-protein coupled receptor protein signaling pathway (GO:0008277)	131	9	.31	28.91	2.42E-07
adenylate cyclase-inhibiting G-protein coupled receptor signaling pathway (GO:0007193)	75	5	.18	28.06	9.22E-03
regulation of epidermal growth factor receptor signaling pathway (GO:0042058)	83	5	.20	25.35	1.51E-02
regulation of ERBB signaling pathway (GO:1901184)	96	5	.23	21.92	3.05E-02
adenylate cyclase-modulating G-protein coupled receptor signaling pathway (GO:0007188)	154	7	.37	19.13	7.38E-04
activation of MAPK activity (GO:0000187)	147	6	.35	17.18	1.23E-02
G-protein coupled receptor signaling pathway, coupled to cyclic nucleotide second messenger (GO:0007187)	181	7	.43	16.28	2.18E-03
regulation of calcium ion transport (GO:0051924)	228	7	.54	12.92	1.01E-02
regulation of MAP kinase activity (GO:0043405)	336	9	.80	11.27	8.09E-04
positive regulation of MAP kinase activity (GO:0043406)	263	7	.62	11.20	2.57E-02
regulation of metal ion transport (GO:0010959)	356	8	.85	9.46	1.65E-02
regulation of protein serine/threonine kinase activity (GO:0071900)	492	9	1.17	7.70	1.90E-02
G-protein coupled receptor signaling pathway (GO:0007186)	1207	22	2.87	7.67	7.76E-11
regulation of MAPK cascade (GO:0043408)	698	10	1.66	6.03	4.24E-02
negative regulation of cell communication (GO:0010648)	1265	14	3.01	4.66	8.26E-03
negative regulation of signaling (GO:0023057)	1269	14	3.02	4.64	8.58E-03
positive regulation of catalytic activity (GO:0043085)	1661	18	3.95	4.56	1.86E-04
positive regulation of molecular function (GO:0044093)	1978	19	4.70	4.04	4.52E-04
regulation of catalytic activity (GO:0050790)	2511	22	5.97	3.69	1.28E-04
system process (GO:0003008)	1844	16	4.38	3.65	2.81E-02
regulation of molecular function (GO:0065009)	3386	26	8.05	3.23	4.41E-05
regulation of cell communication (GO:0010646)	3363	25	7.99	3.13	2.11E-04
regulation of signaling (GO:0023051)	3413	25	8.11	3.08	2.86E-04
regulation of signal transduction (GO:0009966)	3076	22	7.31	3.01	4.90E-03
regulation of response to stimulus (GO:0048583)	4038	25	9.60	2.61	8.17E-03
signal transduction (GO:0007165)	4932	28	11.72	2.39	6.36E-03
signaling (GO:0023052)	5323	30	12.65	2.37	1.88E-03
cell communication (GO:0007154)	5414	30	12.86	2.33	2.80E-03
