# Supplementary material for: Genomic prediction of relapse in recipients of allogeneic haematopoietic stem cell transplantation
Source: Leukemia. 2018 Aug 8;33(1):240–8. doi: 10.1038/s41375-018-0229-3 (PMC6326954; doi:10.1038/s41375-018-0229-3)
Supplement: Supplementary file 2 — Supplementary Methods [file 41375_2018_229_MOESM2_ESM.docx]

Supplementary Methods

**Acquisition and HLA typing of patient samples**

The study cohort was composed of 161 HSCT patients with HLA matched sibling donor. 160 of the patients had relapse status information available and 151 were diagnosed with a malignant disease.

In defining relapse, both hematological and molecular relapses were taken into account. Routine response evaluation (bone marrow morphology with MRD analyses and/or imaging studies) was carried out at three months interval for the first two years post HSCT, and thereafter in case of clinical suspicion of relapse. In lymphoma cases histological confirmation of relapse was required. Thus, relapse was defined as recurrence of disease detected by aforementioned methods at any time point after HSCT.

The study was approved by the Ethics Committees of Helsinki University Central Hospital and Turku University Central Hospital, and the Finnish National Supervisory Authority for Welfare and Health (Valvira; http://www.valvira.fi/web/en/front-page). Informed consent was asked when possible. For some patients the consent could not be asked and in these cases the consent was given by the supervisory authority on the patients’ behalf. The general characteristics of the study cohort are presented in Table 1. In summary, 48 recipients underwent allo-HSCT at Helsinki University Hospital during years 2006–2011 and 113 recipients at Turku University Central Hospital during years 2001–2015. The sibling pairs were matched with regard to HLA-A, -B, -C and -DRB1 loci. HLA typing was performed at low-resolution level with rSSO-Luminex technology (Labtype, One Lambda, Inc., USA) and PCR-SSP (Micro SSP™ Generic HLA Class I/II DNA Typing Trays, One Lambda, Inc.; Olerup SSP® genotyping, Olerup SSP AB, Sweden). High-resolution HLA typing was performed with AlleleSEQR PCR/Sequencing kits (Atria Genetics, USA).

**Sequencing and variant calling**

Next generation sequencing (NGS) was performed at the McGill Genome Centre, McGill University, Montreal, Canada using a custom capture panel targeting whole exome, full MHC region and immune cell active regulatory regions.^1^ The targeted active regulatory regions in CD3^+^, CD3^+^ cord blood, CD4^+^, CD8^+^, CD14^+^, CD19^+^, CD20^+^, CD34^+^, CD56^+^, Th1, Th2, and Th17 cells were selected according to DNAseI hypersensitive sites (DHS) data from the ENCODE and NIH Roadmap Epigenomics projects.^1^ The sequencing was performed using Illumina HiSeq 2000 instrument. The generated reads were aligned to the GRCh37/hg19 reference genome and processed with Genotype Analysis Tool Kit (GATK) v3.2-2 HaplotypeCaller tool as described previously^1^, producing aligned BAM, gVCF and VCF formatted files. The base quality score recalibration and SNP and INDEL discovery was performed using GATK v3.6-0 VariantRecalibrator and ApplyRecalibration tools^2,3^ according to the GATK Best Practices protocols.^4^ The *ts_filter_level* parameter was set to 99.0. The data was further filtered using hard cutoff values for the genotype quality value (GQ)>18. The cutoff values were chosen based on maximizing genotype similarities between duplicated samples; the similarities of two duplicates were compared for each chromosome over varying values of DP and GQ while recording the proportion of discarded variants. The VCF-formatted sequence data was managed using R v3.3.3 (ref. 5) with the library seqminer v5.3 (ref. 6). After quality filtering, variants with minor allele frequency (MAF) below 5% were excluded. The variants were annotated with snpEff v4.2 (ref. 7) using default configuration with genome build GRCh37.75, the custom sequencing panel genomic intervals, and references *NHEK, CD4, GM12878, K562, NH-A, IMR90, HUVEC, HSMM*, and *HMEC*. The GATK tool CombineVariants with the parameter *genotypeMergeOptions* set to *REQUIRE_UNIQUE* was used to merge samples into a single VCF file. Positions not having variants relative to the reference genome were assumed to be the same as reference in the merged and filtered VCF. The GATK runs were performed with Oracle Java v1.8.

**ImmunoChip genotyping**

The cohort and genotyping has been described in detail previously^8^. In brief, the genotyping was performed using Immunochip (Illumina, USA) array, comprising 196524 variants, in 2013, at FIMM Technology Centre, Helsinki, Finland. The DNA samples were extracted from white blood cell fraction of peripheral blood samples. The autosomal genotype data was imputed with IMPUTE2 using 1000 Genomes Phase 3 as a phased reference panel. Pre-filtering of the variants and samples was completed by excluding individuals with missing genotype >3%, variants with minor allele frequency (MAF) <1%, variants with missing data rate >5%, and variants having Hardy–Weinberg equilibrium P-value <0.00001. Post-imputation filtering excluded variants having IMPUTE2 INFO-field measure of the observed statistical information <0.5. After post-imputation filtering, 5041081 variants were included.

**Association tests**

Plink v1.90b3u/v1.90b4.1 (www.cog-genomics.org/plink/1.9/)^9^ was used in SNP/INDEL association tests and related operations. The genotype VCF file was converted to biallelic format with the Plink function *biallelic-only*. Population structure principal component analysis was performed with Plink by first LD-pruning the genotype data using the function *indep-pairwise* with parameter value *50 5 0.8*, and PCA was thereafter computed with the command *pca var-wts*. The output was analysed with R v3.3.3 (5). The principal components were plotted against their eigenvalues to visually identify a threshold where the explained variance reaches a stable level.

Available clinical metadata comprised variables known prior to determining relapse status, i.e. batch, hospital, transplantation date, diagnosis, graft type, conditioning regimen, sex, donor age, recipient age, transplantation sex direction (e.g. male-to-female) and cytomegalovirus (CMV) status. Categorical variables were transformed into numerical format with the R function *model.matrix*. Diagnosis was divided into seven categories: ALL, AML, CLL, MDS, MM, NHL and Other. The ‘Other’ category consisted of various diagnoses, i.e. CMML, DLBCL, TPLL, MF, mantle cell lymphoma, myeloma, Mb Hodgkin, CML, bphenotypic AL, follicular lymphoma and mastocytosis. The variables and included principal components were tested for collinearity using Pearson’s correlation in R. After removing collinear variables, the remaining clinical variables and principal components were tested for association with relapse status with logistic regression using function *glm* in R.

The initial filtering step by genetic association against relapse status was performed with logistic regression using the Plink command *logistic genotypic* with donor age, diagnosis and graft type as covariates. The regression test considered two genetic models, *viz*. additive and dominant/recessive effect. The test was run by systematically leaving one sample out in each iteration, thus producing an individual association output file for each subset of samples.

**Machine learning modeling**

The relapse status of left-out samples was predicted by training a Random forest^10^ classifier model implemented in the R library ranger v0.7.0 (ref. 11) using the same data folds generated in association testing as described above. The model was trained with probabilistic mode, tree number of 2500 and permutated importance. Weights in the model were set according to the proportions cases and controls in each training dataset (e.g. case weights = 1–(relapsed/all)). The features used as predictors were selected by association *p*-value threshold of <0.001 from each training set. The same features were then selected from the sample that was not included in the training set to predict the outcome using the fitted model. The procedure was repeated over all the training sets, producing a leave-one-out cross validation (LOOCV) prediction error estimate whereby feature selection and modeling were both performed within the LOOCV folds. Variable importances were recorded within each LOOCV fold. The general study setup is shown by Figure 1. The training was also performed in the same way by including all clinicial covariates together with genetic variants, and by including the clinical covariates only.

**Evaluation of predictive performance**

The predictive performance of the model was estimated by comparing the distributions of LOOCV predictions between the true relapsed and non-relapsed groups , and by calculating receiver operating characteristic (ROC) and area under the ROC curve (AUC) values for the predictions. The *p*-value between the relapse/no relapse prediction distributions was calculated with one-sided Mann-Whitney test. ROC and AUC values and AUC power at alpha level 0.01 to estimate the type II error were calculated using the R library pROC v1.9.1 (ref. 12) with default settings. The AUC 95% confidence intervals were computed with 2000 stratified bootstrap replicates. The R library ROCR v1.0-7 (ref. 13) was used for calculating odds ratio (defined as (TP*TN)/(FP*FN)) over the range of predicted values. The results were plotted with R v3.3.3.

**Variant ranking**

The permutation-based variable importance metric for the Random forest model was computed within the LOOCV folds and plotted over the folds. Another permutation-based metric using all the samples was computed with the R library Boruta v5.2.0 (ref. 14) with default settings. The Boruta result was averaged over 20 iterations and recoded into numerical format (i.e. 0, 1, 2). Correlation between the two metrics was calculated. Variables having an average Boruta score >1 and LOOCV importance distribution >0 were considered important.

**Variant annotation**

The top predictive variants were examined with ENSEMBL GRCh37.p13 browser and the R library biomaRt v2.30.0 (ref. 15) to determine their possible localization to any gene. The list of genes associating with the top predictive variants (Table 2) was queried against a number of public databanks. ToppGene (https://toppgene.cchmc.org/)^16^ tool ToppFun with databases Pubmed and Interaction (Nov 2017 version) was employed with default settings to analyse the publication topics and interaction partners of the genes. Interactions with Benjamini-Hochberg adjusted p-value at <0.05 were considered significant. The obtained significant interaction partner gene list was functionally annotated with Gene Ontology (GO) v1.2 using PANTHER (http://pantherdb.org/tools)^17^ overrepresentation test (release 20170413) with GO database release 2017-10-23 and all *H.sapiens* genes as a reference. Within the PANTHER GO analysis, the GO Biological Process Complete data was used for annotating the gene list. Bonferroni adjusted p-values <0.05 were considered significant. The top variant gene list was also interrogated against m RNA expression in blood cancer using EMBL-EBI Expression Atlas (https://www.ebi.ac.uk/gxa/genes)^18^ Pan-Cancer Analysis of Whole Genomes – Blood gene expression subset. Another mRNA database used was the Human Protein Atlas (https://www.proteinatlas.org)^19^ cell line dataset, from which blood cancer cell lines (REH, MOLT-4, RPMI-8226, U-266/70, Karpas-707, U-266/84, THP-1, NB-4, HMC-1, HL-60, HEL) expression data were included. PubMed was queried with the search term “((leukemia) OR lymphoma) OR myeloma” and abstracts of the results were downloaded and searched with the Linux command line tool *grep* to find matches against the gene list. Furthermore, cancer gene databases allOnco v3 (https://www.bushmanlab.org/links/genelists)^20-24^, Tsgene v2.0 (https://bioinfo.uth.edu/TSGene/download.cgi)^25^, COSMIC v82 (https://cancer.sanger.ac.uk/cosmic)^26^, and CCGD (https://ccgd-starrlab.oit.umn.edu/download.php)^27^ were queried for presence of the top genes. The data were managed, analysed and plotted with R v3.3.3.

**Replication**

To evaluate the results with an independent set of patients, cohorts of 258 Finnish and 265 Spanish HSCT patients with a sibling donor genotyped with microarray were analysed. None of these patients were included in the primary discovery cohort. The cohorts and genotyping have been described previously in detail.^8^ The top predictive genetic variants from the discovery cohort were matched to the imputed IC SNPs to extract the available genotypes for analysis. Variants missing genotype in the majority of subjects were removed to eliminate excess missing values. The found SNPs in the order of numbers of missing genotypes are given in Supplementary Table 1. The variants remaining after removing missing genotypes were subjected to LOOCV based Random forest modeling and prediction with the *mtry* parameter set to the same value as the number of SNPs. The data were analysed with different numbers of variants removed to estimate the robustness of the approach to variant removal.

A second independent Finnish cohort was analysed to further evaluate the predictive capacity of the top variants in the same population. This cohort initially consisted of 127 patients with a sibling HSCT donor. The ethical approvals and informed consent described above apply to this cohort as well. All recipients underwent allogeneic HSCT at Helsinki or Turku University Hospital transplantation centers, Finland, between years 2004 and 2016. Two patients were excluded before analysis on grounds of diagnosis (aplastic anemia and Mb Wald). The most common diagnoses were AML (33%), ALL (17%), MM (15%) and MDS (12%). GvHD prevention and other treatment procedures were comparable to the discovery cohort. HLA typing and DNA sample collection and processing were performed as described above for the first replication cohort. The used genotyping platform was Infinium® ImmunoArray-24 v2.0 (Illumina), comprising 253702 markers. Genotyping and data preprocessing were performed as described above for the first replication cohort. Ungenotyped markers were imputed using Beagle v5.0 (ref. 28) with default settings, and probabilistic genotype estimates from the imputation were used in downstream analyses. As with the first replication cohort, the imputed markers were quality filtered to exclude unreliable markers. Imputation was considered low-quality if allele frequencies significantly deviated from the discovery cohort. Two filtering thresholds based on genotype standard deviation were used: <0.3 leaving the variants rs599115, rs1065375, rs910500, rs11585739, rs4846913, rs1432297, rs35194171, rs35741374, rs1177205, rs1177206, rs1177207, rs750026, rs750027, rs842625, rs842631, rs11678404, rs3848858, rs9405201, rs17309827, rs10456096 and <0.2 leaving the same variants plus rs4367936, rs35927656, rs12543811. The The predictive modeling was performed as described above for the first replication cohort. The results are shown by Figure 2c.

**Analysis of individual diagnoses**

AML patients (n=55) were extracted from the full dataset and analysed as a separate group to evaluate whether limiting to a single diagnosis affects predictive performance. AML was selected because it was the largest diagnosis group. The analysis was carried out as described above for discovery and replication cohorts, except for LOOCV variant selection step where the diagnosis covariate was not used. The results are given in Supplementary Figure 7. To further define the impact of individual diagnoses on predictive performance, the original discovery cohort result consisting of heterogeneous malignancies was factorised into diagnosis components. The diagnosis groups were defined as described above for covariate analysis. The results are given in Supplementary Figure 8.

**Supplementary references**

1. Morin A, Kwan T, Ge B, Letourneau L, Ban M, Tandre K*, et al*. Immunoseq: the identification of functionally relevant variants through targeted capture and sequencing of active regulatory regions in human immune cells. *BMC Med Genomics* 2016; **9**: 59,016-0220-7.

2. McKenna A, Hanna M, Banks E, Sivachenko A, Cibulskis K, Kernytsky A*, et al*. The Genome Analysis Toolkit: a MapReduce framework for analyzing next-generation DNA sequencing data. *Genome Res* 2010; **20**: 1297-303.

3. DePristo MA, Banks E, Poplin R, Garimella KV, Maguire JR, Hartl C*, et al*. A framework for variation discovery and genotyping using next-generation DNA sequencing data. *Nat Genet* 2011; **43**: 491-8.

4. Van der Auwera GA, Carneiro MO, Hartl C, Poplin R, Del Angel G, Levy-Moonshine A*, et al*. From FastQ data to high confidence variant calls: the Genome Analysis Toolkit best practices pipeline. *Curr Protoc Bioinformatics* 2013; **43**: 11.10.1-33.

5. R Core Team. R: A language and environment for statistical computing. 2017; **3.3.3**.

6. Zhan X, Liu DJ. SEQMINER: An R-Package to Facilitate the Functional Interpretation of Sequence-Based Associations. *Genet Epidemiol* 2015; **39**: 619-23.

7. Cingolani P, Platts A, Wang le L, Coon M, Nguyen T, Wang L*, et al*. A program for annotating and predicting the effects of single nucleotide polymorphisms, SnpEff: SNPs in the genome of Drosophila melanogaster strain w1118; iso-2; iso-3. *Fly (Austin)* 2012; **6**: 80-92.

8. Hyvarinen K, Ritari J, Koskela S, Niittyvuopio R, Nihtinen A, Volin L*, et al*. Genetic polymorphism related to monocyte-macrophage function is associated with graft-versus-host disease. *Sci Rep* 2017; **7**: 15666,017-15915-3.

9. Chang CC, Chow CC, Tellier LC, Vattikuti S, Purcell SM, Lee JJ. Second-generation PLINK: rising to the challenge of larger and richer datasets. *Gigascience* 2015; **4**: 7,015-0047-8. eCollection 2015.

10. Breiman L. Random Forests. *Machine Learning* 2001; **45**: 5-32.

11. Wright MN, Ziegler A. ranger: A Fast Implementation of Random Forests for High Dimensional Data in C++ and R. *J STAT SOFTW* 2017; **77**.

12. Robin X, Turck N, Hainard A, Tiberti N, Lisacek F, Sanchez JC*, et al*. pROC: an open-source package for R and S+ to analyze and compare ROC curves. *BMC Bioinformatics* 2011; **12**: 77,2105-12-77.

13. Sing T, Sander O, Beerenwinkel N, Lengauer T. ROCR: visualizing classifier performance in R. *Bioinformatics* 2005; **21**: 3940-1.

14. Kursa MB, Rudnicki WR. Feature Selection with the Boruta Package. *J STAT SOFTW* 2010; **36**.

15. Durinck S, Spellman PT, Birney E, Huber W. Mapping identifiers for the integration of genomic datasets with the R/Bioconductor package biomaRt. *Nat Protoc* 2009; **4**: 1184-91.

16. Chen J, Bardes EE, Aronow BJ, Jegga AG. ToppGene Suite for gene list enrichment analysis and candidate gene prioritization. *Nucleic Acids Res* 2009; **37**: W305-11.

17. Mi H, Huang X, Muruganujan A, Tang H, Mills C, Kang D*, et al*. PANTHER version 11: expanded annotation data from Gene Ontology and Reactome pathways, and data analysis tool enhancements. *Nucleic Acids Res* 2017; **45**: D183-9.

18. Petryszak R, Keays M, Tang YA, Fonseca NA, Barrera E, Burdett T*, et al*. Expression Atlas update--an integrated database of gene and protein expression in humans, animals and plants. *Nucleic Acids Res* 2016; **44**: D746-52.

19. Uhlen M, Fagerberg L, Hallstrom BM, Lindskog C, Oksvold P, Mardinoglu A*, et al*. Proteomics. Tissue-based map of the human proteome. *Science* 2015; **347**: 1260419.

20. Vogelstein B, Papadopoulos N, Velculescu VE, Zhou S, Diaz LA,Jr, Kinzler KW. Cancer genome landscapes. *Science* 2013; **339**: 1546-58.

21. Futreal PA, Coin L, Marshall M, Down T, Hubbard T, Wooster R*, et al*. A census of human cancer genes. *Nat Rev Cancer* 2004; **4**: 177-83.

22. Akagi K, Suzuki T, Stephens RM, Jenkins NA, Copeland NG. RTCGD: retroviral tagged cancer gene database. *Nucleic Acids Res* 2004; **32**: D523-7.

23. Sjoblom T, Jones S, Wood LD, Parsons DW, Lin J, Barber TD*, et al*. The consensus coding sequences of human breast and colorectal cancers. *Science* 2006; **314**: 268-74.

24. Huret JL, Minor SL, Dorkeld F, Dessen P, Bernheim A. Atlas of genetics and cytogenetics in oncology and haematology, an interactive database. *Nucleic Acids Res* 2000; **28**: 349-51.

25. Zhao M, Kim P, Mitra R, Zhao J, Zhao Z. TSGene 2.0: an updated literature-based knowledgebase for tumor suppressor genes. *Nucleic Acids Res* 2016; **44**: D1023-31.

26. Forbes SA, Beare D, Boutselakis H, Bamford S, Bindal N, Tate J*, et al*. COSMIC: somatic cancer genetics at high-resolution. *Nucleic Acids Res* 2017; **45**: D777-83.

27. Abbott KL, Nyre ET, Abrahante J, Ho YY, Isaksson Vogel R, Starr TK. The Candidate Cancer Gene Database: a database of cancer driver genes from forward genetic screens in mice. *Nucleic Acids Res* 2015; **43**: D844-8.

28. Browning BL, Browning SR. Genotype Imputation with Millions of Reference Samples. *Am J Hum Genet* 2016; **98**: 116-26.
