# Supplementary material for: Genomic prediction of relapse in recipients of allogeneic haematopoietic stem cell transplantation
Source: Leukemia. 2018 Aug 8;33(1):240–8. doi: 10.1038/s41375-018-0229-3 (PMC6326954; doi:10.1038/s41375-018-0229-3)
Supplement: Supplementary file 3 — Supplementary Figures [file 41375_2018_229_MOESM3_ESM.pdf]

Supplementary Figures

a

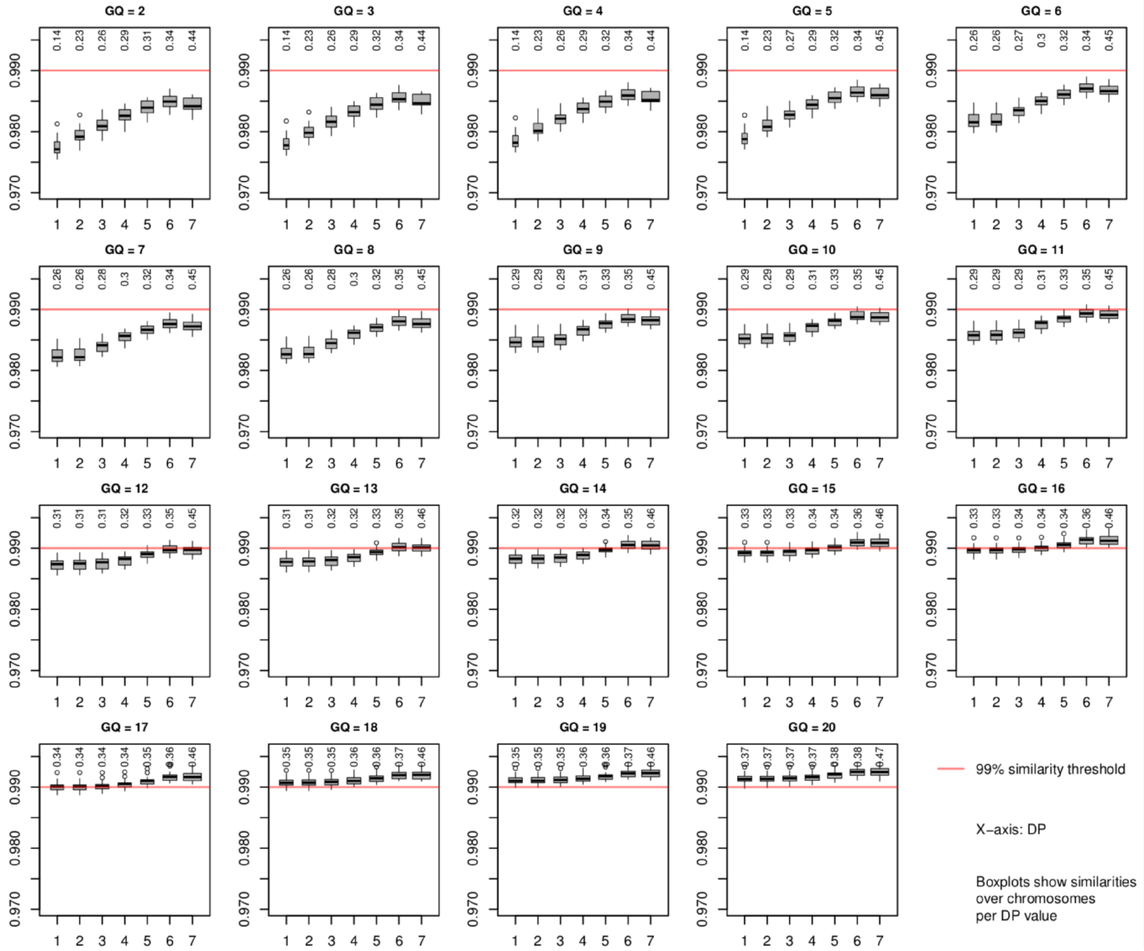

b

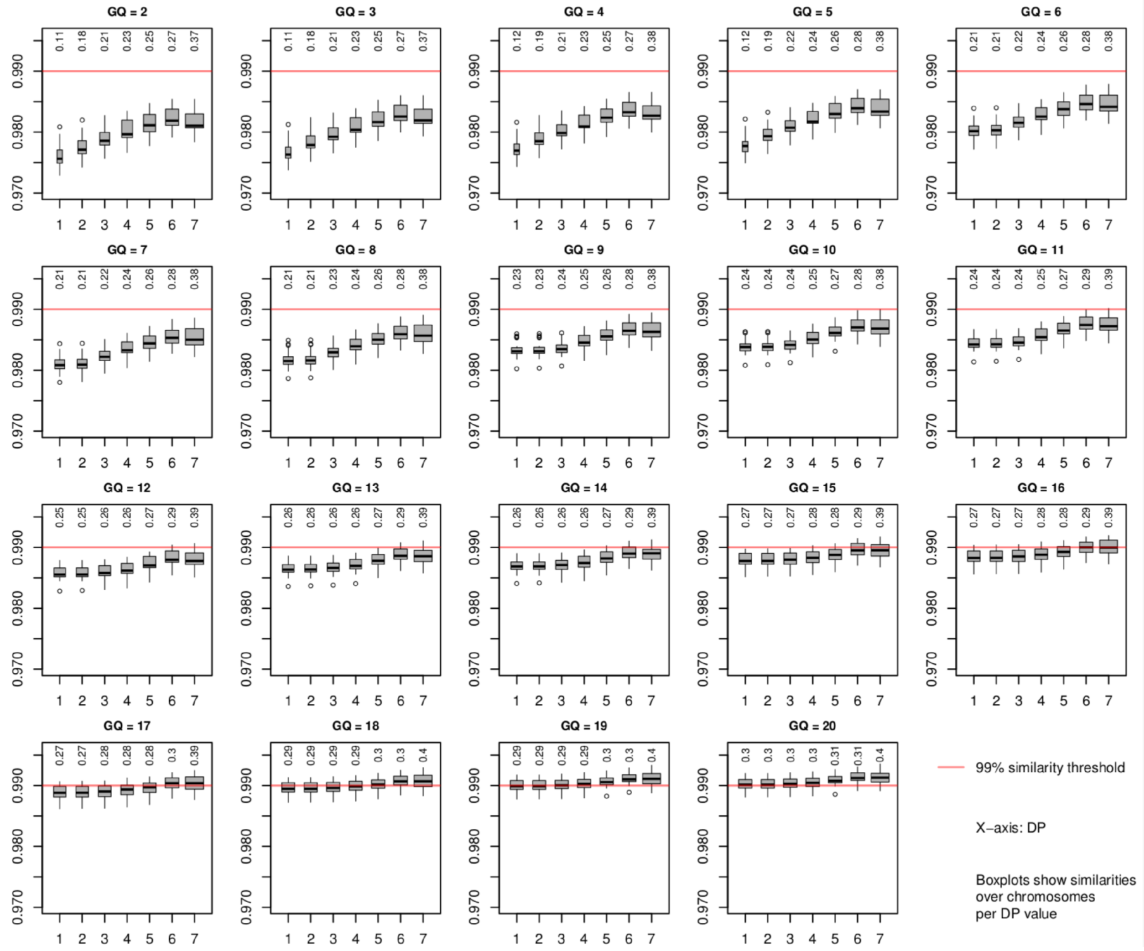

Supplementary Figure 1. Variant filtering by GQ and DP parameter hard cutoff in two duplicated subjects (**a, b**). The separate plot panels show the impact of varying the value of GQ parameter on the genotype similarity (y-axis) between the duplicates. Each boxplot shows the similarity distribution over chromosomes 1-22. The x-axes of the plots show the applied DP value cutoff. The numbers above boxplots indicate the proportion of variants that are discarded due to the applied DP value at a particular GQ cutoff value.

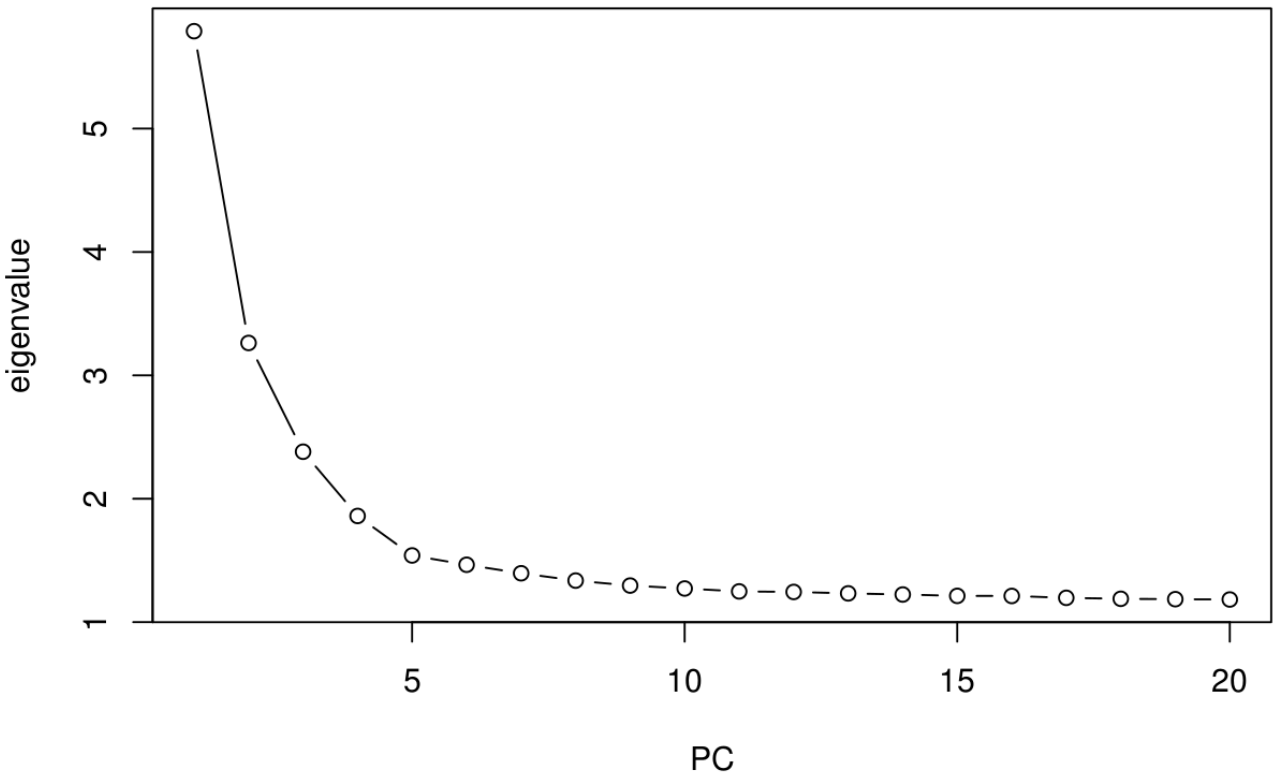

Supplementary Figure 2. Principal component eigenvalues vs. the components.

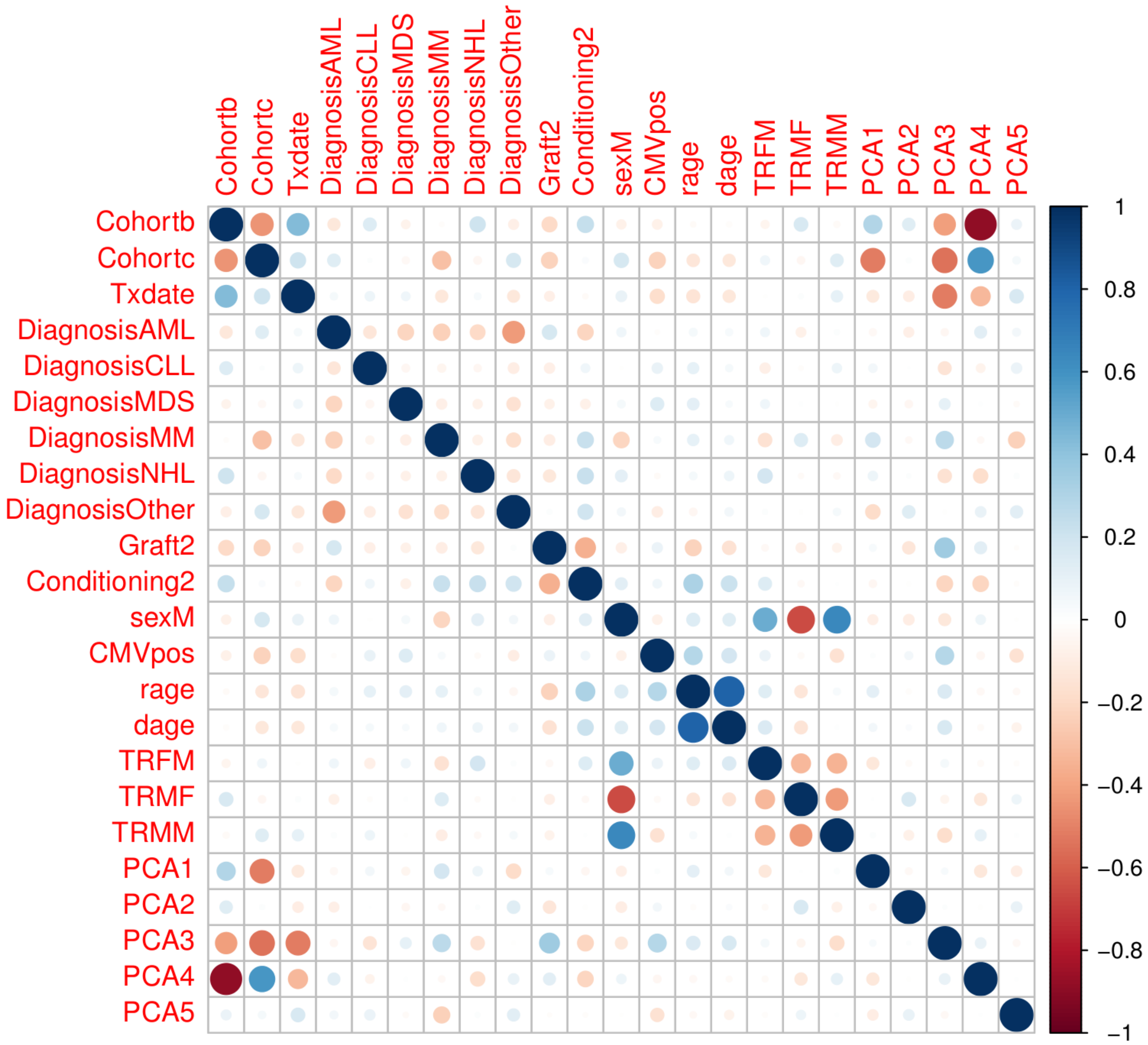

Supplementary Figure 3. Covariate correlation matrix.

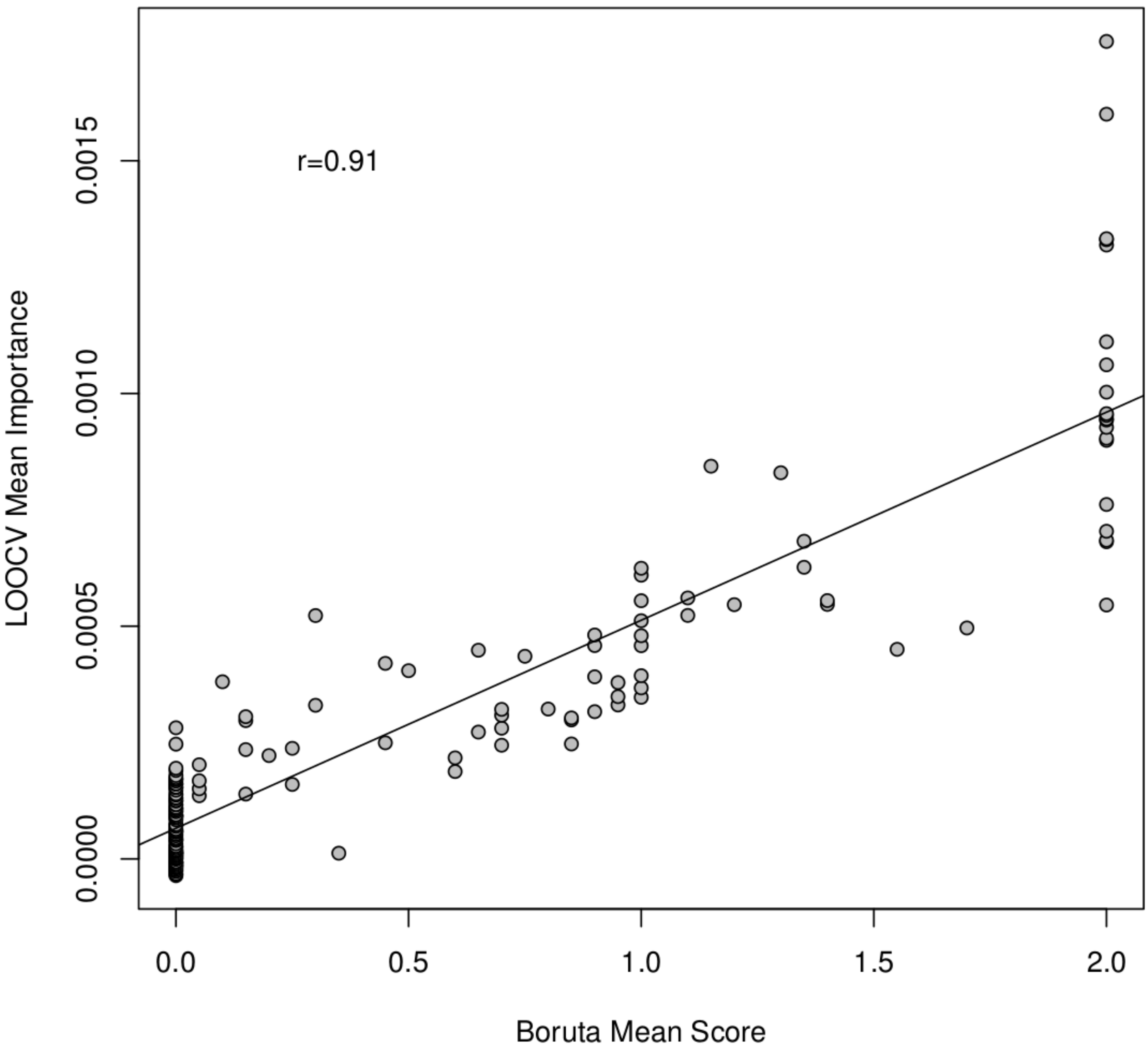

Supplementary Figure 4. orrelation between two variant ranking metrics. On the x-axis, the mean Boruta score, and on the y-axis, the mean LOOCV importance.

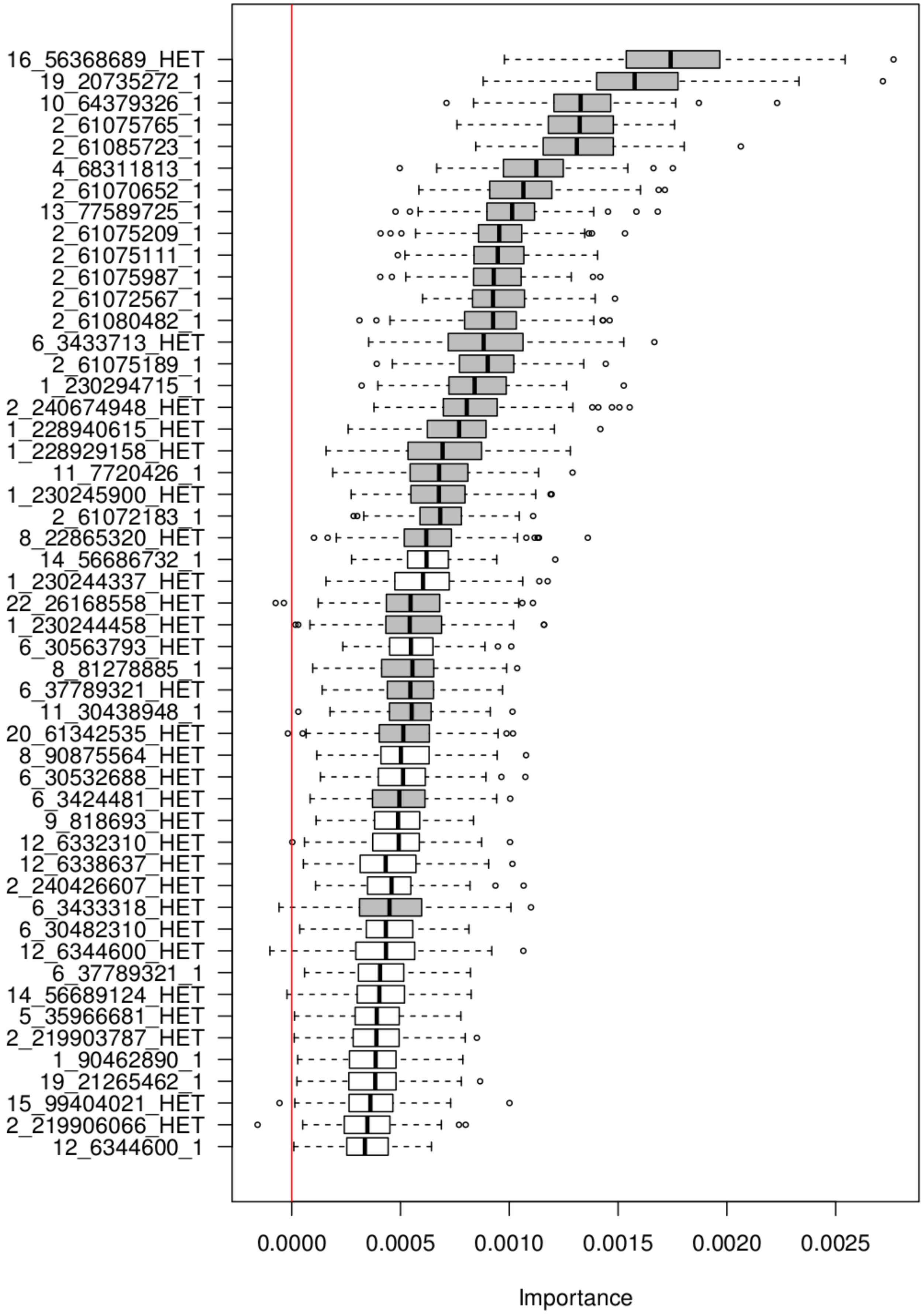

Supplementary Figure 5. Variant ranking plot. The x-axis shows the variable importance value. The boxplots on the y-axis show the importance distribution over the LOOCV folds for the top 50 variants in order of importance. The variant names follow the *chromosome_position_model* format, where in the model part “1” indicates additive and “HET” indicates dominant/recessive genetic model. The grey filling of the boxplots shows the variants having a mean Boruta score over 1.

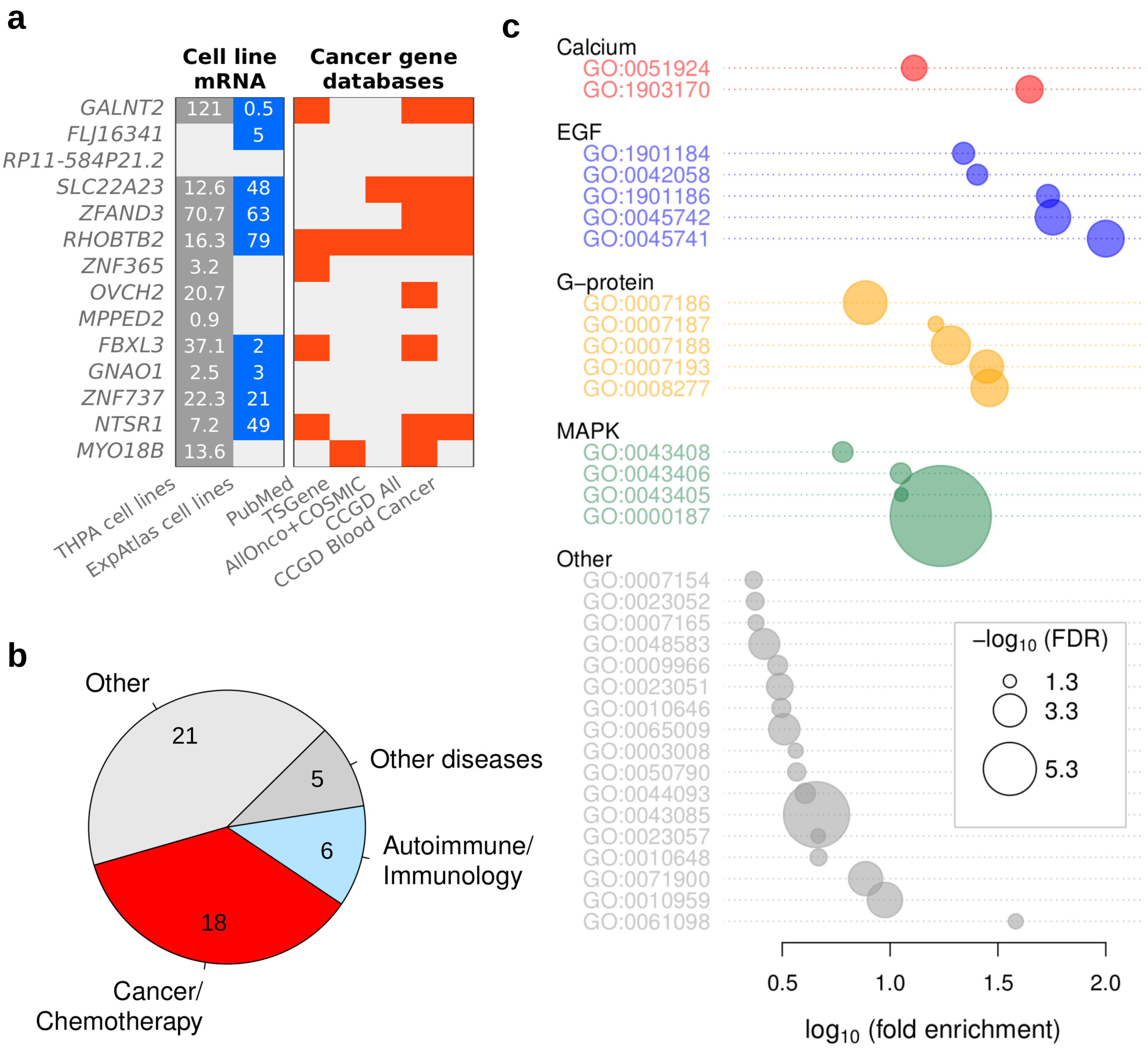

Supplementary Figure 6. Functional characterization of the genes associated with the top predictive variants. (a) Representation of the genes in public cancer-related databases. Colour indicates presence while light grey indicates absence per given data resource. The numbers in the first two columns indicate mRNA expression magnitude measured in TPM (transcripts per million) in blood cancer cell lines available in the Human Protein Atlas database and in the Expression Atlas database. The "Database" columns shows the presence of the genes in cancer gene databases. Here, "PubMed" indicates PubMed abstracts restricted to blood cancer keywords. (b) Topics of significantly (FDR<0.05) enriched PubMed literature citations for the genes according to ToppGene analysis. The "Other disease" category indicates diseases other than autoimmune or cancer, and the "Other" category indicates a study subject other than any disease. Details of the PubMed enrichment analysis results are given in Supplementary Table 4. (c) Statistically significant enrichment of the genes and their protein-protein interaction partners in the Gene Ontology Biological Process functional categories. Calcium and G-protein signaling, epidermal growth factor (EGF) and MAP kinase pathways present the highest fold enrichment (x-axis). The size of the spheres indicates the enrichment FDR value. Both the interaction partners and the GO enrichment results are selected at FDR<0.05. Details of the interaction results are given in Supplementary Table 5 and the GO enrichment results in Supplementary Table 6.

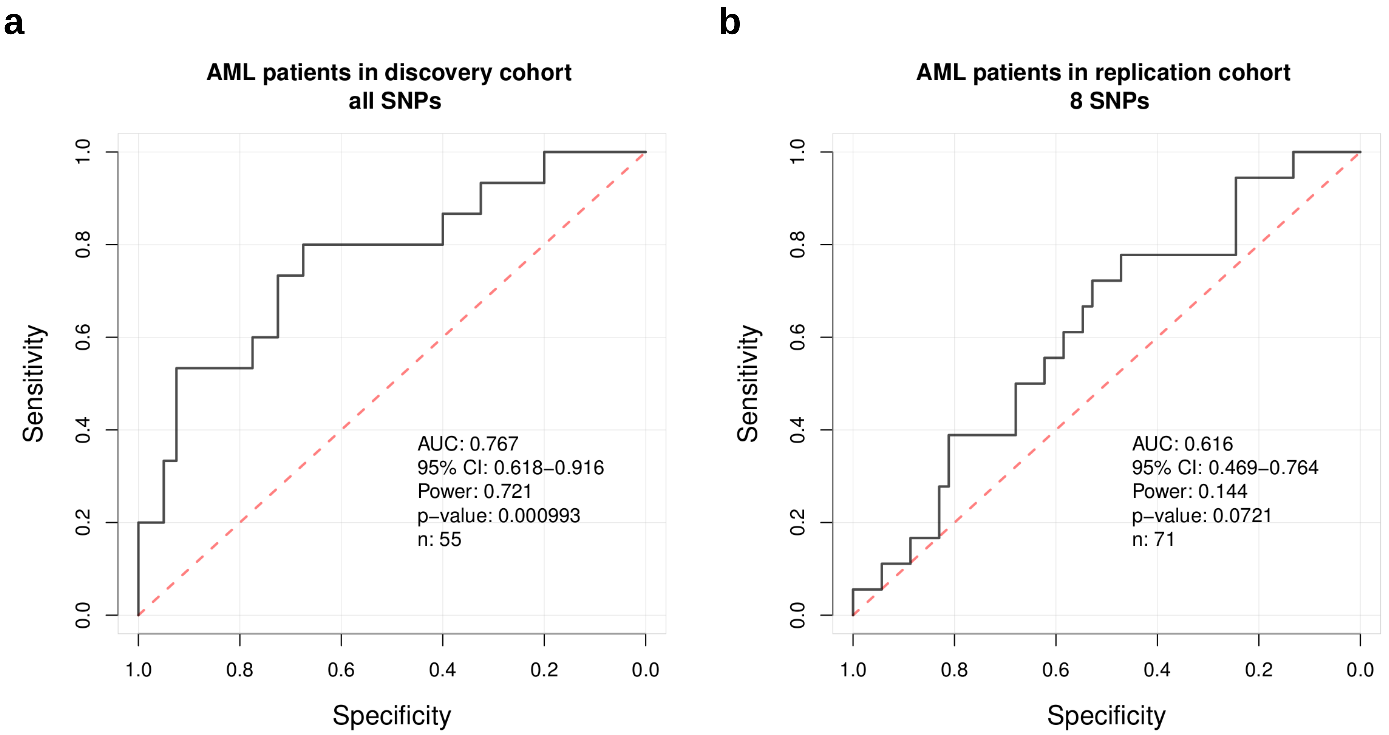

c

CHR	Position	refsnp ID	ENSEMBL Gene ID	Gene symbol	Gene description
12	6091164	rs216868	ENSG00000110799	*VWF*	von Willebrand factor
17	53478675	rs3744308	ENSG00000108960	*MMD*	monocyte to macrophage differentiation associated
4	74944834	rs13128896	NA	*NA*	Intergenic
1	74663814	rs1412825	ENSG00000254685	*FPGT*	fucose-1-phosphate guanylyltransferase
2	204305093	rs3814365	ENSG00000173166	*RAPH1*	Ras association (RalGDS/AF-6) and pleckstrin homology domains 1
19	46972967	rs2288916	ENSG00000182013	*PNMA8A*	PNMA family member 8A
2	96687427	rs200675258	ENSG00000186281	*GPAT2*	glycerol-3-phosphate acyltransferase 2, mitochondrial
3	69435706	rs6769509	ENSG00000114541	*FRMD4B*	FERM domain containing 4B
11	39767016	rs1158736	NA	*NA*	Intergenic
1	223815575	rs34187970	ENSG00000203697	*CAPN8*	calpain 8
4	70584083	rs13138435	ENSG00000173597	*SULT1B1*	sulfotransferase family 1B member 1
10	81839472	rs7085612	ENSG00000230091	*TMEM254-AS1*	TMEM254 antisense RNA 1
8	90914432	rs10088155	ENSG00000207359	*RNU6-925P*	RNA, U6 small nuclear 925, pseudogene
11	73345882	rs10793063	NA	*NA*	Intergenic
1	230197780	rs75879691	ENSG00000143641	*GALNT2*	polypeptide N-acetylgalactosaminyltransferase 2
9	33541056	rs7035916	ENSG00000230453	*ANKRD18B*	ankyrin repeat domain 18B
12	52966390	rs11170176	ENSG00000170484	*KRT74*	keratin 74
16	69360265	rs35318292	ENSG00000132612	*VPS4A*	vacuolar protein sorting 4 homolog A
6	26370572	rs34878490	ENSG00000186470	*BTN3A2*	butyrophilin, subfamily 3, member A2
1	234659605	rs71638498	ENSG00000231768	*LINC01354*	long intergenic non-protein coding RNA 1354

Supplementary Figure 7. Relapse analysis limited to AML patients. (**a**) LOOCV ROC/AUC results of the discovery cohort based on genome-wide feature selection, and (**b**) results on Finnish replication cohort based on eight top SNPs present on the imputated Immunocip platform. (**c**) List of top predictive variants from the discovery cohort in order of importance.

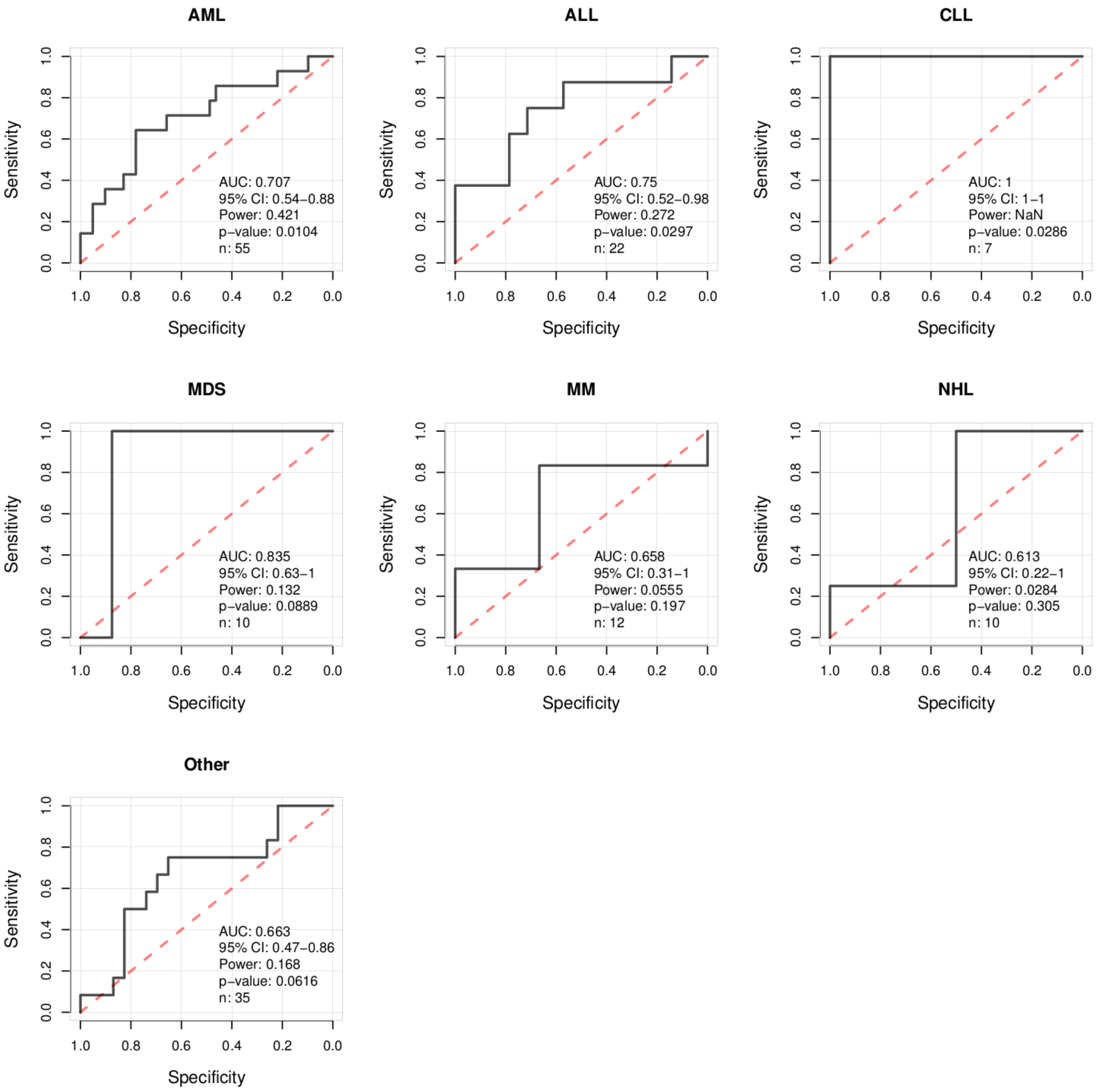

Supplementary Figure 8. LOOCV ROC/AUC results of the full discovery cohort factorized into diagnosis components. The 'Other' group is defined in the Supplementary Methods.
